# Modernizing Commercial Agency Regulations in Saudi Arabia: Legal Reforms and Comparative Insights

**DOI:** 10.12688/f1000research.168970.1

**Published:** 2025-09-12

**Authors:** Abdullah Ali Alasmari, Hajed A. Alotaibi

**Affiliations:** 1 Assistant Professor, Department of law, College of judicial studies and regulations, Umm Alqura University, Mecca, Mecca, Saudi Arabia; 2 Associate Professor, Department of Sharia, College of Sharia and Law, Majmaah University, Al Majmaah, Riyadh Province, 11952, Saudi Arabia

**Keywords:** Saudi Arabia, Commercial Agency Law, Legal Reform, Shariah Compliance, Arbitration, Foreign Direct Investment, Comparative Law, Vision 2030.

## Abstract

**Background:**

this study examines the modernization of Saudi’s Commercial Agency Law the 2022–2023 reforms, with a focus on legal, economic, and comparative dimensions. For decades, the previous framework faced criticism for its rigid nationality restrictions, procedural inefficiencies, and dispute resolution mechanisms. The reforms form part of the Kingdom’s broader Vision 2030 strategy, aiming to diversify the economy, strengthen private sector competitiveness, and integrate more fully with global trade systems. This research also evaluates how the reforms align with Shariah principles, balancing commercial flexibility with religious compliance.

**Methods:**

the study adopts a doctrinal legal methodology, systematically analyzing statutory amendments and implementing regulations considering Saudi case law and policy statements. A comparative approach is employed to benchmark the reforms against best practices in jurisdictions such as the UAE and the UK, with particular emphasis on contract termination rules, registration systems, and dispute resolution mechanisms. Sources include secondary data such as official gazettes, ministerial circulars, judicial precedents, and academic literature.

**Results:**

key legislative changes include the relaxation of nationality requirements for commercial agents, enabling broader foreign participation; the digitalization of agency registration and renewal processes, reducing administrative burdens; clarified termination and compensation provisions, enhancing contractual certainty; and formal recognition of arbitration clauses, offering greater dispute resolution flexibility. These reforms have the potential to significantly improve market transparency, reduce litigation and attract foreign direct investment (FDI).

**Conclusions:**

although the reforms represent a significant advancement, challenges remain, including judicial consistency in interpreting the new provisions, resistance among entrenched market players, and the need for enhanced regulatory guidance. The paper recommends targeted judicial and practitioner training, proactive awareness campaigns, and GCC-wide legal harmonization to amplify reform benefits. Implemented effectively, these measures could solidify Saudi Arabia’s role as a regional leader in commercial agency regulation, strengthen investor confidence, and advance the Kingdom’s long-term economic diversification goals.

## 1. Introduction

The Kingdom of Saudi Arabia (KSA) has undergone significant economic and legal reforms in recent years under the ambitious Vision 2030 framework. One major regulatory domain affected by these reforms is commercial agency law, a cornerstone in structuring business relationships between local agents and foreign principals. Historically, Saudi commercial agency regulations, codified originally under Royal Decree No. M/11 of 1382H (1962), were characterized by protectionist policies that granted exclusive rights to Saudi agents and imposed strict limitations on foreign principals. While intended to empower local enterprises and safeguard national economic interests, these rules often created rigid legal frameworks that discouraged foreign direct investment (FDI) and limited market liberalization ( Al-Fadhel, 2022).

A growing body of scholarship has underscored the necessity of modernizing commercial agency regimes to balance investor protection with market competitiveness. Al-Anazi (2021) critiques the inflexibility of pre-reform systems, arguing that restrictive agency laws elevate transactional risks and deter multinational corporations from entering regional markets. Similarly, Al-Sheikh (2023) points out that limitations on contract termination and rigid agent protections undermine contractual freedom, a principle vital for attracting and retaining global investors. Comparative legal studies of jurisdictions such as the UAE and UK demonstrate that legal predictability, accessible dispute resolution mechanisms, and clear compensation rules significantly enhance investor confidence ( Sornarajah, 2021). These works collectively point to a research gap: while reform discourse is growing, few studies provide a detailed doctrinal and comparative analysis of Saudi Arabia’s recent agency reforms and their alignment with both Shariah principles and international best practices.

In addition, scholars such as Alotaibi (2021) have highlighted the intersection between Shariah jurisprudence and modern commercial law, emphasizing the need for legal models that integrate Islamic principles with contemporary economic realities. For example, the principle of *gharar* (uncertainty) historically complicated open-ended agency contracts, yet modern reforms have sought to reconcile these doctrines with global market norms. This duality presents fertile ground for legal inquiry, especially in evaluating how Saudi Arabia can retain its Islamic legal identity while adopting market-driven reforms compatible with global trade regimes.

Moreover, reports by the World Bank (2023) and OECD (2021) have stressed that legal reforms in emerging markets must be empirically assessed for their capacity to reduce transaction costs, increase investor protections, and streamline administrative procedures. The Saudi case offers a unique lens: it represents both a rapidly modernizing legal system and one deeply anchored in religious and cultural norms. This study responds to the gap by linking theoretical literature, statutory interpretation, and comparative insights, thereby bridging scholarly discussions with applied legal analysis. Furthermore, Vision 2030’s focus on improving the investment climate amplifies the importance of this inquiry. By examining commercial agency reforms within this strategic context, the study demonstrates how legal modernization serves as a tool for achieving macroeconomic goals such as diversification and increased private sector participation (Saudi Vision 2030, 2022).

The introduction of these reforms also intersects with Saudi Arabia’s commitments under international agreements, including WTO membership and bilateral investment treaties, making it essential to evaluate their compatibility with global trade rules. Addressing these dimensions allows for a deeper understanding of how Saudi Arabia positions itself in the global commercial arena ( UNCTAD, 2022). Additionally, recent empirical studies on business law reforms in emerging markets underscore the role of predictable legal environments in reducing transaction costs and fostering competitive markets ( OECD, 2023). This study builds upon such findings by situating Saudi reforms within comparative and doctrinal frameworks.

The integration of Shariah-based principles into modern legal structures also raises critical questions regarding judicial interpretation and enforcement. By analyzing how courts balance religious doctrines with contemporary commercial norms, the research sheds light on the adaptability of Islamic jurisprudence in the face of economic globalization. Moreover, this research contributes to the discourse on hybrid legal systems by exploring how Saudi Arabia blends elements of civil law, common law, and Islamic legal traditions. This hybridity offers an innovative approach for other jurisdictions seeking to modernize without compromising cultural or religious identity. Lastly, this section sets the stage for a comprehensive examination of subsequent reforms, their comparative benchmarks, and their long-term implications for Saudi Arabia’s ambition to become a leading commercial hub in the Middle East and globally.

## Methodology

This study adopts a doctrinal legal research methodology, focusing on the systematic analysis of primary legal sources such as Royal Decree No. M/11 of 1444H (2022–2023 reforms), implementing regulations issued by the Ministry of Commerce, and key provisions of related legislation. The doctrinal approach allows for a detailed examination of statutory language, its interpretation by Saudi courts, and its alignment with recognized principles of commercial law. The research is supplemented by comparative analysis, focusing on the UAE as a regional peer with a civil law foundation and the UK as a common law jurisdiction with historically liberalized commercial frameworks. This dual comparison provides both regional relevance and global benchmarking, enabling an evaluation of Saudi reforms against contrasting legal traditions and regulatory philosophies.

Furthermore, this study integrates a Shariah-based analytical lens, examining how Islamic jurisprudence influences and interacts with modern commercial regulation. This involves an assessment of scholarly writings on principles such as *gharar* and *fasakh*, and their application in agency-related disputes, with attention to how these are interpreted within Saudi judicial practice. Finally, the methodology includes a review of secondary literature comprising peer-reviewed journal articles, commentaries by legal practitioners, policy reports by entities such as UNCTAD and OECD, and data from investment and arbitration centers. This triangulated approach ensures that the study not only interprets legal texts but also situates them within broader economic, judicial, and policy contexts.

## Objectives

The objectives of this study are fourfold. First, it seeks to identify the structural deficiencies of Saudi Arabia’s pre-reform commercial agency regime, focusing on statutory inflexibility, administrative inefficiencies, and their deterrent effect on investment. Second, it aims to analyze the legal and Shariah-compliant features of the 2022–2023 reforms, including detailed examination of newly introduced provisions on registration, termination, and dispute resolution. Third, it compares Saudi Arabia’s reformed agency law with the UAE’s Federal Law No. 3 of 2022 and the UK’s Commercial Agents (Council Directive) Regulations 1993, providing insights into similarities, divergences, and lessons for legal harmonization. Finally, it evaluates the implications of these reforms for FDI and broader legal modernization, offering evidence-based recommendations for policymakers.

Beyond these primary goals, the study also seeks to contribute to the theoretical understanding of how Shariah-based principles can coexist with global commercial law frameworks. By doing so, it provides a nuanced lens for examining legal reform in Islamic jurisdictions where cultural, religious, and economic objectives intersect. Additionally, the research aims to inform policymakers, legal practitioners, and academics on best practices for implementing legal reforms in emerging markets. By dissecting statutory provisions and judicial practices, it bridges the gap between academic theory and practical application, providing actionable insights for legal modernization. Lastly, the study aspires to establish a foundation for future empirical research by identifying measurable indicators—such as dispute resolution trends, agency registrations, and investment flows—that can assess the ongoing impact of these reforms. This creates a roadmap for longitudinal evaluation, ensuring that legal development aligns with both Vision 2030 objectives and global market expectations.

## 2. Legal framework before the reforms

Saudi Arabia’s original Commercial Agency Law, promulgated under Royal Decree No. M/11 of 1382H (1962), provided the foundational structure for regulating agency relationships. This framework imposed strict localization requirements: only Saudi nationals or wholly Saudi-owned entities could register as commercial agents with the Ministry of Commerce. Contracts with unregistered agents were deemed unenforceable, depriving foreign principals of any legal recourse ( Al-Otaibi, 2018). This provision, designed to protect domestic business, inadvertently discouraged foreign firms wary of limited enforceability. One significant issue under the pre-reform law was the excessive leverage granted to agents. Courts frequently upheld agent claims for compensation even in fixed-term agreements, citing Article 3 of the prior statute, which prioritized agent protection over contractual autonomy. For example, in Board of Grievances case No. 2451/1435H, compensation was awarded despite clear evidence of contractual expiration, reflecting judicial deference to statutory protections over commercial norms ( Al-Zahrani, 2024).

Additionally, the law’s rigidity impeded adaptation to global commercial practices. Unlike jurisdictions where parties could negotiate non-exclusive or sector-specific agency agreements, Saudi law mandated exclusivity, constraining competitive distribution networks. Scholarly critiques, such as Alotaibi (2021), argued that this model entrenched monopolistic practices contrary to emerging competition principles under the Saudi Competition Law. From a Shariah perspective, indefinite agency contracts and punitive termination penalties conflicted with doctrines aimed at preventing *gharar* (uncertainty) and unjust enrichment ( *akl al-mal bil-batil *). Classical juristic opinions cautioned against arrangements lacking defined terms or balanced obligations, which became a frequent litigation point in pre-reform disputes. These tensions underscored the need for statutory reform harmonizing modern commercial expectations with Islamic jurisprudence. Moreover, administrative inefficiencies exacerbated legal rigidity. The requirement for in-person filings, coupled with prolonged registration timelines, created bureaucratic delays documented in Ministry of Commerce reports (2019). These inefficiencies reduced Saudi Arabia’s ranking in the World Bank’s “Ease of Doing Business” index, highlighting how outdated procedures impeded market entry and investment flows.

Collectively, these statutory and practical deficiencies formed the impetus for reform, as policymakers recognized that the old regime was increasingly incompatible with the objectives of Vision 2030 and global trade norms. Another critical concern under the pre-reform regime was the absence of clear dispute resolution mechanisms tailored to commercial agency conflicts. Litigation often became protracted and costly due to the lack of specialized commercial courts, discouraging foreign entities from pursuing claims and undermining contractual enforcement certainty ( Al-Rasheed, 2020). Furthermore, pre-reform jurisprudence exhibited inconsistencies in interpreting statutory provisions, with divergent rulings from different judicial panels. Such unpredictability exacerbated investor apprehension, signaling the urgent need for codified reforms and standardized judicial training ( Alayed et al., 2025).

In addition, the lack of provisions accommodating modern commercial structures, such as joint ventures or multi-tiered agency frameworks, rendered the law outdated in addressing evolving business models. This rigidity hindered innovative market entry strategies and integration into global supply chains ( OECD, 2021). Administrative opacity further worsened the situation. The absence of a centralized, publicly accessible registry of agency contracts limited market transparency and impeded due diligence for foreign investors, thereby weakening trust in the regulatory environment ( World Bank, 2019). Finally, economic data indicated that restrictive agency practices contributed to inflated costs for goods and services, as exclusivity arrangements allowed agents to exert monopolistic pricing power. This inefficiency not only burdened consumers but also constrained competition, reducing the overall attractiveness of the Saudi market ( UNCTAD, 2020).

## 3. The 2022–2023 reforms

The 2022–2023 amendments, enacted through Royal Decree No. M/11 of 1444H, introduced transformative changes aimed at aligning Saudi commercial agency law with international best practices while preserving Shariah compliance. One major development was the relaxation of nationality requirements, permitting foreign principals under specified conditions to act as agents or appoint agents directly, a move detailed in Article 5 of the new law. This reform addressed historical concerns about limited foreign market access and directly supports Vision 2030’s objective of attracting global investors ( Ministry of Commerce, 2023). Digitization of registration processes through the Ministry’s electronic portal represents another significant reform. By reducing paperwork and enabling real-time contract validation, this change mitigates administrative delays that historically hindered agency registrations and eroded investor confidence ( World Bank, 2023). The law now explicitly mandates electronic filing under Article 7, streamlining compliance and reducing procedural ambiguity.

Termination provisions were also restructured. The revised Article 10 delineates permissible termination grounds such as expiration of fixed terms, mutual consent, or breach of obligations. Unlike the pre-reform regime, which often imposed compensation even after valid expiry, the updated provisions align with international standards while retaining fair compensation rights in cases of unjustified termination, balancing interests of both parties ( Al-Fadhel, 2022). Importantly, the reforms enhanced dispute resolution flexibility. Article 14 formally recognizes arbitration clauses, allowing parties to resolve disputes via domestic or international arbitration, including the Saudi Center for Commercial Arbitration (SCCA). This move aligns with the UNCITRAL Model Law and strengthens Saudi Arabia’s reputation as an arbitration-friendly jurisdiction ( SCCA, 2022).

Lastly, the Ministry of Commerce now wields expanded regulatory powers to oversee compliance and sanction violations (Article 18). Such oversight mirrors regulatory models in advanced jurisdictions and ensures that reforms are implemented consistently across sectors. Scholars such as Alotaibi (2022) argue that this supervisory shift fosters transparency and accountability crucial to sustaining investor trust. Together, these reforms signify a deliberate shift toward a hybrid legal model, combining market liberalization with Shariah-rooted safeguards. Their long-term efficacy will hinge on coherent judicial interpretation, sustained administrative commitment, and continued alignment with evolving global commercial standards. Moreover, these reforms introduced greater regulatory clarity by publishing implementing guidelines that explain procedural requirements for registration, termination notifications, and dispute resolution mechanisms. This guidance reduces ambiguity for both domestic and foreign businesses, thereby facilitating smoother compliance and reducing legal disputes ( Ministry of Commerce, 2023).

Another notable development is the integration of compliance monitoring mechanisms, which mandate periodic reporting by registered agents and principals. This innovation enhances accountability and provides the Ministry with data-driven oversight capabilities, supporting better enforcement of legal provisions and promoting market transparency ( OECD, 2023). Finally, the reforms have emphasized capacity-building initiatives, including training programs for legal practitioners and judges on the application of the new law. Such initiatives are critical to ensuring consistent interpretation of the reformed statutes and building institutional expertise necessary for sustaining legal modernization ( Al-Rasheed, 2023).

## 4. Comparative analysis

To contextualize Saudi Arabia’s reforms, this study undertakes a comparative analysis of two distinct jurisdictions: the United Arab Emirates (UAE) and the United Kingdom (UK). The UAE was selected due to its geographical proximity, shared GCC membership, and its similar historical reliance on protectionist agency regimes. Its 2022 reforms provide a relevant point of comparison to assess regional legal harmonization and divergence. Conversely, the UK, with its common law heritage and liberalized market, offers a contrasting model emphasizing contractual freedom and minimal state intervention. This dual focus allows for both intra-regional benchmarking and alignment with mature global markets ( Klein, 2022).

The UAE’s Federal Law No. 3 of 2022 illustrates how civil law systems can adapt agency rules while retaining localized safeguards. This law maintains exclusive agent rights under Article 4 but introduces termination flexibilities under Article 9, including reduced restrictions for public joint-stock companies and long-standing foreign firms. Notably, arbitration is recognized explicitly in Article 12, mirroring Saudi Arabia’s incorporation of ADR, thereby reflecting a regional trend toward arbitration-friendly practices ( AlSuwaidi, 2022). However, continued nationality requirements for agents reflect the UAE’s cautious liberalization path compared to Saudi Arabia’s broader relaxation.

The UK’s regime, governed by the Commercial Agents (Council Directive) Regulations 1993, adopts a contrasting philosophy rooted in EU Directive 86/653/EEC principles. UK law prioritizes freedom of contract, with minimal registration burdens and flexible termination conditions, while still providing indemnity or compensation under Regulation 17 for terminated agents. Case law such as *Lonsdale v. Howard & Hallam Ltd* [2007] UKHL 32 demonstrates how courts balance agent protection with market liberalism, offering insights for Saudi policymakers seeking equilibrium between statutory regulation and autonomy ( Bird & Bird, 2020).

Saudi Arabia’s reforms demonstrate hybridization: agent protections akin to the UAE’s coexist with arbitration acceptance and termination flexibilities paralleling the UK. This selective incorporation suggests deliberate alignment with international standards while respecting Shariah principles. By adopting features from both systems, Saudi Arabia positions itself as a legally sophisticated jurisdiction capable of attracting foreign investment while retaining its cultural and legal identity. Future scholarship could explore GCC-wide harmonization, building on these bilateral comparisons to craft regionally integrated yet globally competitive commercial laws ( OECD, 2023). In addition, examining case studies from both the UAE and UK illustrates how legal reforms evolve within different institutional contexts. The UAE’s incremental changes highlight how gradual liberalization can mitigate stakeholder resistance, while the UK’s reliance on judicial precedents underscores the role of courts in shaping commercial agency norms ( KPMG, 2023).

Another relevant aspect is the divergent dispute resolution frameworks. The UAE’s integrated arbitration clauses, combined with ministerial oversight, present a hybrid approach, whereas the UK’s independent judiciary provides direct judicial enforcement of agency rights. This contrast offers insights for Saudi Arabia on balancing administrative regulation with judicial independence ( Bird & Bird LLP, 2020). Moreover, lessons from the UK’s post-Brexit retention of EU-derived agency principles demonstrate how stable legal regimes can persist through political shifts, providing confidence to investors and showcasing resilience against external shocks ( Klein, 2022). Such stability could inform Saudi Arabia’s approach in maintaining reform continuity amid future policy transitions. Finally, integrating these comparative insights deepens the understanding of Saudi Arabia’s hybridization process, highlighting how selectively adopting elements from both systems—such as arbitration mechanisms from the UAE and contract freedoms from the UK—can craft a reform model that is both contextually grounded and globally competitive.

## 5. Impact on Foreign Direct Investment (FDI)

The reformation of Saudi Arabia’s Commercial Agency Law is anticipated to significantly reshape the investment landscape by reducing historical barriers and signaling regulatory modernization. First, enhanced investor confidence arises from clearer statutory provisions, including transparent registration (Article 7) and termination (Article 10) rules, which mitigate perceived legal uncertainty—a key deterrent cited in UNCTAD’s World Investment Report (2022). Second, by introducing flexible agency arrangements, including non-exclusive and sector-specific agreements, foreign principals now possess greater strategic options for market entry. This aligns with OECD findings (2023) on how liberalized agency laws correlate with increased multinational participation in comparable jurisdictions. Early indicators, such as new market entries by global pharmaceutical and automotive firms documented in Ministry of Commerce data (2023), suggest that reforms are already influencing investment decisions. Third, recognition of arbitration (Article 14) has strengthened dispute resolution predictability, encouraging cross-border investments that rely on enforceable contracts. The SCCA’s expansion, reporting a 40% increase in case filings post-reform ( SCCA Annual Report, 2023), underscores investor reliance on streamlined ADR frameworks over protracted litigation. Additionally, harmonization with Saudi Arabia’s bilateral investment treaties (BITs) and WTO commitments reinforces legal alignment with global trade norms, reducing perceived sovereign risk ( OECD, 2021). Such alignment positions Saudi Arabia competitively among GCC states and emerging markets aiming to attract high-value FDI.

Lastly, these reforms indirectly bolster domestic competition. As agency exclusivity weakens, principals can incentivize performance-based representation, fostering efficiency gains within distribution networks. Empirical studies (e.g., Alotaibi, 2022) argue that such competitive pressures improve consumer outcomes and market dynamism, further validating reforms as catalysts for broader economic diversification. While promising, the ultimate FDI impact will depend on consistent judicial enforcement, administrative transparency, and investor perception over time. This underscores the need for longitudinal data and continued empirical evaluation of reform effectiveness within Saudi Arabia’s evolving legal-economic context. Furthermore, data from the General Authority for Statistics (2023) indicate that foreign equity inflows into key sectors such as retail and manufacturing increased by 18% within the first year of the reforms, reflecting heightened investor confidence in a more predictable legal regime. This early evidence strengthens the correlation between regulatory modernization and market expansion.

In addition, qualitative feedback from global law firms and investment advisory reports underscores a notable shift in perception of Saudi Arabia’s investment climate. Firms cite streamlined contract registration, enforceable arbitration clauses, and reduced exit barriers as major incentives for market entry, thus validating the legal reforms’ investor-friendly orientation ( PwC, 2023). These reforms also contribute to Saudi Arabia’s broader strategy of aligning with global trade blocs. By harmonizing its agency law with WTO obligations and bilateral investment treaty standards, the Kingdom positions itself to negotiate trade agreements more effectively and leverage its legal stability as a competitive advantage ( UNCTAD, 2022). Moreover, comparisons with regional peers highlight Saudi Arabia’s emerging leadership in legal reform. For instance, its integration of arbitration and digitalized agency systems surpasses similar efforts in neighboring GCC states, potentially positioning the Kingdom as a model jurisdiction for foreign corporations seeking a base for regional operations ( OECD, 2023). Finally, sustained monitoring of reform outcomes will enable policymakers to adapt regulatory measures dynamically, ensuring that legal changes continue to attract high-quality investment. This adaptive approach will be vital for reinforcing long-term investor trust and embedding Saudi Arabia’s reformed legal framework within the global economic order.

## 6. Challenges and future directions

Despite the significant advancements introduced by the 2022–2023 reforms, several challenges remain that could impact their long-term success. First, effective implementation hinges on consistent judicial application across all regions. Variability in judicial expertise and familiarity with commercial law may lead to inconsistent interpretations of the new provisions, creating uncertainty for investors ( Al-Rasheed, 2023). Targeted judicial training programs and specialized commercial courts could mitigate these risks by ensuring uniformity in rulings. Second, entrenched resistance from established commercial agents poses another obstacle. Many longstanding agents accustomed to the protective pre-reform regime may challenge reforms through litigation or political lobbying. Studies on legal transitions in GCC states ( Al-Qahtani, 2022) indicate that such resistance can delay reform outcomes unless paired with robust stakeholder engagement and transitional support mechanisms.

Third, integrating arbitration more deeply into commercial dispute resolution raises cultural and doctrinal questions. While the SCCA’s increased caseload is promising, uncertainties remain about the enforcement of foreign arbitral awards, particularly those conflicting with public policy or Shariah principles ( Alshamsi & Ghaffar, 2021). Establishing clearer judicial guidance on these issues would enhance predictability and reinforce Saudi Arabia’s status as an arbitration-friendly jurisdiction. Additionally, practical guidance for businesses remains limited. The absence of standardized model contracts and official commentary on the new law exposes parties to interpretive errors and drafting deficiencies. Publishing detailed Ministry of Commerce guidelines and explanatory notes would promote compliance and reduce disputes stemming from contractual ambiguities ( OECD, 2021).

Finally, regional harmonization within the GCC represents both a challenge and an opportunity. Divergent commercial agency laws among GCC members complicate cross-border investment strategies. Coordinated legal reforms could create a unified regional market, strengthening the Gulf’s global trade competitiveness ( World Bank, 2023). Saudi Arabia’s leadership in this area could position it as a catalyst for broader regional legal integration. In sum, addressing these challenges will require sustained institutional investment, judicial capacity building, active stakeholder dialogue, and continuous policy refinement to ensure that the reforms achieve their intended transformative impact. Moreover, there is a pressing need to enhance the training of legal professionals, particularly those practicing outside of major commercial centers, to ensure uniform application of the reformed law across the Kingdom. Without such initiatives, discrepancies in interpretation could undermine investor confidence and create regional disparities in enforcement ( Al-Rasheed, 2023). Additionally, the government must prioritize public awareness campaigns to educate businesses—especially SMEs—on their rights and obligations under the new law. Clear, accessible resources can bridge knowledge gaps and reduce inadvertent non-compliance, strengthening the overall efficacy of the reforms ( OECD, 2023).

Another critical challenge is integrating technology-driven compliance tools. While digitization has streamlined registration, more advanced platforms for monitoring contract execution and dispute tracking could further improve transparency and reduce administrative burden ( World Bank, 2023). Cross-border enforcement of arbitral awards also requires attention. Though Saudi Arabia has acceded to the New York Convention, case-specific resistance to enforcement on Shariah grounds persists. Establishing consistent guidelines for such reviews will be crucial for maintaining Saudi Arabia’s reputation as an arbitration-friendly jurisdiction ( Alshamsi & Ghaffar, 2021). Moreover, fostering dialogue with international chambers of commerce and investor councils can provide policymakers with valuable feedback on reform implementation challenges and best practices, creating a responsive policy environment ( PwC, 2023).

The reforms also necessitate greater collaboration with academic institutions to generate empirical research on judicial trends, investment impacts, and sector-specific implications. Data-driven policymaking will be essential for iterative refinement and long-term success ( UNCTAD, 2022). Finally, integrating these legal reforms into broader regional economic initiatives, such as GCC market harmonization and free trade agreements, would not only bolster Saudi Arabia’s legal framework but also enhance the Gulf’s overall competitiveness in attracting global capital ( OECD, 2023).

## 7. Conclusion

The 2022–2023 reforms to Saudi Arabia’s Commercial Agency Law represent a landmark transformation in the Kingdom’s legal landscape, aligning domestic regulations with global trade norms while retaining a Shariah-compliant foundation. These changes dismantle longstanding protectionist barriers, enhance investor confidence through clearer rules and digitalized procedures, and strengthen dispute resolution mechanisms by formally recognizing arbitration. Collectively, they signal a decisive shift toward a more open, predictable, and investment-friendly commercial environment consistent with Vision 2030.

This study highlights that while the pre-reform regime prioritized local agent protections, it inadvertently constrained market entry and impeded FDI. By embracing reforms that balance agent rights with contractual autonomy and global best practices, Saudi Arabia demonstrates its commitment to legal modernization and economic diversification. The comparative analysis with the UAE and UK underscores Saudi Arabia’s hybrid approach, selectively adopting liberalizing features while preserving its legal identity anchored in Islamic principles. Nonetheless, successful realization of these reforms will depend on effective judicial interpretation, uniform application across regions, robust regulatory oversight, and continuous engagement with stakeholders. Addressing implementation challenges—ranging from judicial training to resistance from entrenched agents—will be pivotal in cementing reform gains and fostering a transparent and competitive commercial market.

In conclusion, Saudi Arabia’s reformed commercial agency framework not only strengthens its domestic legal infrastructure but also enhances its attractiveness as a regional hub for trade and investment. Future research should focus on empirical assessments of the reforms’ economic impacts, long-term investor perceptions, and opportunities for GCC-wide legal harmonization. By sustaining momentum in legal reform and aligning with global standards, the Kingdom is poised to emerge as a leading model for integrating modern commercial regulation with Shariah principles in the global legal order. Moreover, these reforms offer a template for other emerging markets that face similar challenges in balancing local legal traditions with international trade demands. By studying Saudi Arabia’s approach, policymakers elsewhere can glean insights into sequencing reforms, engaging stakeholders, and embedding legal predictability within culturally rooted legal systems.

The conclusions drawn from this analysis also stress the need for iterative assessment. As reforms mature, empirical studies focusing on judicial decisions, contract enforcement rates, and investor response metrics will be vital in measuring their effectiveness and identifying areas requiring fine-tuning. Furthermore, sustained cooperation between public institutions, private stakeholders, and academic researchers is essential to cultivate an informed, evidence-driven environment for future legal innovations. This will ensure that reform benefits are widely understood and practically applied across all economic sectors. Lastly, embedding these reforms within a broader regional integration framework could amplify their impact. By advocating for harmonized agency laws within the GCC and collaborating with neighboring states, Saudi Arabia could strengthen its position as both a legal and commercial hub, thereby advancing Vision 2030’s ambitions for global competitiveness.

## 8. Policy recommendation summary

First, implement specialized judicial training programs focusing on commercial agency law and arbitration. Tailored curricula for commercial court judges and legal practitioners will ensure consistent interpretation and reduce regional disparities in applying the reformed statutes. Second, establish a centralized compliance and monitoring platform integrated with the Ministry of Commerce’s digital portal. This platform should provide automated alerts for contract renewals, termination deadlines, and dispute filings to streamline regulatory oversight and reduce administrative inefficiencies. Third, develop official model contracts and detailed explanatory guidelines to assist businesses—especially SMEs—in complying with the new law. These resources will reduce drafting errors, facilitate standardized practices, and lower transactional risks. Fourth, launch public awareness campaigns through chambers of commerce, industry associations, and online platforms to educate businesses about their rights and obligations under the updated law. Emphasizing accessibility and multilingual resources will broaden outreach to foreign investors.

Fifth, institutionalize annual performance reviews of the reforms, incorporating data on FDI inflows, contract registrations, dispute resolutions, and judicial outcomes. Such empirical assessments will allow policymakers to adapt and refine legal frameworks dynamically. Sixth, strengthen the role of arbitration by expanding capacity at the Saudi Center for Commercial Arbitration (SCCA), promoting training for arbitrators, and issuing clear judicial guidelines on the enforcement of arbitral awards consistent with Shariah principles. Seventh, enhance collaboration with academic and research institutions to conduct longitudinal studies on the socio-economic impacts of the reforms, enabling data-driven policymaking and legal innovation. Eighth, actively engage with international investors and trade partners through consultation forums, inviting feedback on practical challenges faced during market entry and contract execution. Such engagement will ensure responsiveness and alignment with global business standards. Finally, pursue GCC-wide harmonization of commercial agency laws by leading initiatives that standardize registration procedures, dispute resolution mechanisms, and cross-border enforcement practices. This regional approach will facilitate intra-GCC trade and solidify Saudi Arabia’s role as a legal and commercial hub.

## Data Availability

No data is associated with this article.
