# Modernizing Commercial Agency Regulations in Saudi Arabia: Legal Reforms and Comparative Insights

**DOI:** 10.12688/f1000research.168970.2

**Published:** 2025-11-18

**Authors:** Abdullah Ali Alasmari, Hajed A. Alotaibi

**Affiliations:** 1 Assistant Professor, Department of law, College of judicial studies and regulations, Umm Alqura University, Mecca, Mecca, Saudi Arabia; 2 Associate Professor, Department of Sharia, College of Sharia and Law, Majmaah University, Al Majmaah, Riyadh Province, 11952, Saudi Arabia

**Keywords:** Saudi Arabia, Commercial Agency Law, Legal Reform, Shariah Compliance, Arbitration, Foreign Direct Investment, Comparative Law, Vision 2030.

## Abstract

**Background:**

This study analyzes Saudi Arabia’s 2022–2023 reforms to the Commercial Agency Law through legal, economic, and comparative lenses. The pre-reform regime—marked by rigid nationality limits, procedural burdens, and litigation-prone termination rules—constrained market entry and investor confidence.

**Methods:**

Using a doctrinal approach to statutes and implementing regulations, triangulated with Saudi judicial practice and policy instruments, we benchmark Saudi reforms against the UAE and UK. A Shariah lens clarifies how *gharar* (uncertainty), *fasakh* (rescission), and unjust enrichment ( *akl al-māl bil-bāṭil*) shape agency disputes.

**Results:**

Key changes include more flexible nationality rules, end-to-end digital registration and renewal, clearer termination/compensation standards, and formal recognition of arbitration (including SCCA). Together these measures enhance contractual predictability and lower compliance frictions, with early indications of improved market transparency and investor sentiment.

**Conclusions:**

The reforms signal a hybrid model—liberalization aligned with global practice while preserving a Shariah-grounded identity. Remaining priorities include consistent judicial application, practitioner training, clearer guidance, and GCC coordination. We propose thematically grouped policy steps to consolidate gains and support Vision 2030’s diversification agenda.

## 1. Introduction

The Kingdom of Saudi Arabia (KSA) has undergone significant economic and legal reforms in recent years under the ambitious Vision 2030 framework. One major regulatory domain affected by these reforms is commercial agency law, a cornerstone in structuring business relationships between local agents and foreign principals. Historically, Saudi commercial agency regulations, codified originally under Royal Decree No. M/11 of 1382H (1962), were characterized by protectionist policies that granted exclusive rights to Saudi agents and imposed strict limitations on foreign principals. While intended to empower local enterprises and safeguard national economic interests, these rules often created rigid legal frameworks that discouraged foreign direct investment (FDI) and limited market liberalization ( Al-Fadhel, 2022).

A growing body of scholarship has underscored the necessity of modernizing commercial agency regimes to balance investor protection with market competitiveness. Al-Anazi (2021) critiques the inflexibility of pre-reform systems, arguing that restrictive agency laws elevate transactional risks and deter multinational corporations from entering regional markets. Similarly, Al-Sheikh (2023) points out that limitations on contract termination and rigid agent protections undermine contractual freedom, a principle vital for attracting and retaining global investors. Comparative legal studies of jurisdictions such as the UAE and UK demonstrate that legal predictability, accessible dispute resolution mechanisms, and clear compensation rules significantly enhance investor confidence ( Sornarajah, 2021). These works collectively point to a research gap: while reform discourse is growing, few studies provide a detailed doctrinal and comparative analysis of Saudi Arabia’s recent agency reforms and their alignment with both Shariah principles and international best practices.

In addition, scholars such as Alotaibi (2021) have highlighted the intersection between Shariah jurisprudence and modern commercial law, emphasizing the need for legal models that integrate Islamic principles with contemporary economic realities. For example, the principle of *gharar* (uncertainty) historically complicated open-ended agency contracts, yet modern reforms have sought to reconcile these doctrines with global market norms. This duality presents fertile ground for legal inquiry, especially in evaluating how Saudi Arabia can retain its Islamic legal identity while adopting market-driven reforms compatible with global trade regimes.

Moreover, reports by the World Bank (2023) and OECD (2021) have stressed that legal reforms in emerging markets must be empirically assessed for their capacity to reduce transaction costs, increase investor protections, and streamline administrative procedures. The Saudi case offers a unique lens: it represents both a rapidly modernizing legal system and one deeply anchored in religious and cultural norms. This study responds to the gap by linking theoretical literature, statutory interpretation, and comparative insights, thereby bridging scholarly discussions with applied legal analysis. Furthermore, Vision 2030’s focus on improving the investment climate amplifies the importance of this inquiry. By examining commercial agency reforms within this strategic context, the study demonstrates how legal modernization serves as a tool for achieving macroeconomic goals such as diversification and increased private sector participation (Saudi Vision 2030, 2022).

The introduction of these reforms also intersects with Saudi Arabia’s commitments under international agreements, including WTO membership and bilateral investment treaties, making it essential to evaluate their compatibility with global trade rules. Addressing these dimensions allows for a deeper understanding of how Saudi Arabia positions itself in the global commercial arena ( UNCTAD, 2022). Additionally, recent empirical studies on business law reforms in emerging markets underscore the role of predictable legal environments in reducing transaction costs and fostering competitive markets ( OECD, 2023). This study builds upon such findings by situating Saudi reforms within comparative and doctrinal frameworks.

The integration of Shariah-based principles into modern legal structures also raises critical questions regarding judicial interpretation and enforcement. By analyzing how courts balance religious doctrines with contemporary commercial norms, the research sheds light on the adaptability of Islamic jurisprudence in the face of economic globalization. Moreover, this research contributes to the discourse on hybrid legal systems by exploring how Saudi Arabia blends elements of civil law, common law, and Islamic legal traditions. This hybridity offers an innovative approach for other jurisdictions seeking to modernize without compromising cultural or religious identity. Lastly, this section sets the stage for a comprehensive examination of subsequent reforms, their comparative benchmarks, and their long-term implications for Saudi Arabia’s ambition to become a leading commercial hub in the Middle East and globally.

**Conceptual framing:** The selective incorporation of foreign rules into Saudi commercial agency law engages classic debates on legal transplants and institutional adaptation. Transplants can accelerate modernization, yet borrowed rules may underperform without fit to local institutions and normative anchors. In a mixed system grounded in Shariah, the risk is not formal incompatibility but interpretive friction unless adjudicative practice and administrative guidance evolve in tandem. This also situates the reforms within law-and-development discussions that weigh globalization’s standard-setting pull against legal particularism and legitimacy.

In this article, we treat Saudi Arabia’s post-2022 regime as a hybridization strategy: targeted liberalization (digitalization, arbitration, termination clarity) is combined with calibrated agent protections to preserve relational stability. We therefore test two implications throughout the paper: (i) whether hybridization increases predictability for foreign principals without eroding doctrinal coherence for domestic actors; and (ii) whether institutional adaptation judicial technique, regulatory guidance, and practitioner capacity mediates the success of transplanted features.

### 1.1 Methodology and sources

This study adopts a doctrinal legal research methodology, focusing on the systematic analysis of primary legal sources such as Royal Decree No. M/11 of 1444H (2022–2023), implementing regulations issued by the Ministry of Commerce, and key provisions of related legislation. The doctrinal approach allows for a detailed examination of statutory language, its interpretation by Saudi courts, and its alignment with recognized principles of commercial law. The research is supplemented by comparative analysis, focusing on the UAE as a regional peer with a civil law foundation and the UK as a common law jurisdiction with historically liberalized commercial frameworks. This dual comparison provides both regional relevance and global benchmarking, enabling an evaluation of Saudi reforms against contrasting legal traditions and regulatory philosophies. Furthermore, this study integrates a Shariah-based analytical lens, examining how Islamic jurisprudence influences and interacts with modern commercial regulation. This involves an assessment of scholarly writings on principles such as *gharar* and *fasakh*, and their application in agency-related disputes, with attention to how these are interpreted within Saudi judicial practice. Finally, the methodology includes a review of secondary literature comprising peer-reviewed journal articles, commentaries by legal practitioners, policy reports by entities such as UNCTAD and OECD, and data from investment and arbitration centers. This triangulated approach ensures that the study not only interprets legal texts but also situates them within broader economic, judicial, and policy contexts.

To anchor the doctrinal and comparative analysis, we explicitly draw on theoretical lenses outlined in the *Introduction*. Legal transplant and institutional-adaptation perspectives guide our reading of why specific features (e.g., arbitration recognition, digital filing, compensation calibration) were selected and how they are likely to function within a *Shariah*-grounded adjudicative culture. This avoids purely descriptive comparison and frames our empirical baselines in the FDI and registration domains. To ensure methodological transparency, the corpus of legal texts analyzed covers the period 2018–2024, capturing both pre-reform and post-reform practice. Inclusion criteria encompassed all circulars and ministerial directives publicly issued under the Ministry of Commerce’s digital portal, as well as secondary literature providing interpretive context. Exclusion criteria omitted unpublished or inaccessible administrative guidance.

The doctrinal analysis proceeds clause-by-clause, quoting operative statutory text where appropriate and interpreting it in light of Shariah maxims (qawāʿid fiqhiyyah) and comparative statutory equivalents. The analysis pays particular attention to how Saudi courts reconcile modern statutory provisions with Shariah principles such as gharar (uncertainty), fasakh (rescission), and akl al-māl bil-bāṭil (unjust enrichment). The comparative dimension employs a purposive sampling of the UAE Commercial Agency Law (Federal Law No. 3 of 2022) and the UK Commercial Agents (Council Directive) Regulations 1993. Comparative evaluation follows a three-step logic: (1) identify statutory parallels, (2) analyze their adaptation to local institutional constraints, and (3) evaluate cross-system transferability. The theoretical framing draws from legal transplant and institutional adaptation theories, explaining why particular reforms were selectively borrowed and how they interact with Saudi Arabia’s Shariah-based adjudicative culture. This allows the paper to move beyond description toward explaining how and why specific features travel and succeed.

### 1.2 Objectives

The objectives of this study are fourfold. First, it seeks to identify the structural deficiencies of Saudi Arabia’s pre-reform commercial agency regime, focusing on statutory inflexibility, administrative inefficiencies, and their deterrent effect on investment. Second, it aims to analyze the legal and Shariah-compliant features of the 2022–2023 reforms, including detailed examination of newly introduced provisions on registration, termination, and dispute resolution. Third, it compares Saudi Arabia’s reformed agency law with the UAE’s Federal Law No. 3 of 2022 and the UK’s Commercial Agents (Council Directive) Regulations 1993, providing insights into similarities, divergences, and lessons for legal harmonization. Finally, it evaluates the implications of these reforms for FDI and broader legal modernization, offering evidence-based recommendations for policymakers.

Beyond these primary goals, the study also seeks to contribute to the theoretical understanding of how Shariah-based principles can coexist with global commercial law frameworks. By doing so, it provides a nuanced lens for examining legal reform in Islamic jurisdictions where cultural, religious, and economic objectives intersect. Additionally, the research aims to inform policymakers, legal practitioners, and academics on best practices for implementing legal reforms in emerging markets. By dissecting statutory provisions and judicial practices, it bridges the gap between academic theory and practical application, providing actionable insights for legal modernization. Lastly, the study aspires to establish a foundation for future empirical research by identifying measurable indicators—such as dispute resolution trends, agency registrations, and investment flows—that can assess the ongoing impact of these reforms. This creates a roadmap for longitudinal evaluation, ensuring that legal development aligns with both Vision 2030 objectives and global market expectations.

## 2. Pre-reform legal framework

Saudi Arabia’s original Commercial Agency Law, promulgated under Royal Decree No. M/11 of 1382H (1962), provided the foundational structure for regulating agency relationships. This framework imposed strict localization requirements: only Saudi nationals or wholly Saudi-owned entities could register as commercial agents with the Ministry of Commerce. Contracts with unregistered agents were deemed unenforceable, depriving foreign principals of any legal recourse ( Al-Otaibi, 2018). This provision, designed to protect domestic business, inadvertently discouraged foreign firms wary of limited enforceability. One significant issue under the pre-reform law was the excessive leverage granted to agents. Courts frequently upheld agent claims for compensation even in fixed-term agreements, citing Article 3 of the prior statute, which prioritized agent protection over contractual autonomy. For example, in Board of Grievances case No. 2451/1435H, compensation was awarded despite clear evidence of contractual expiration, reflecting judicial deference to statutory protections over commercial norms ( Al-Zahrani, 2024).

Additionally, the law’s rigidity impeded adaptation to global commercial practices. Unlike jurisdictions where parties could negotiate non-exclusive or sector-specific agency agreements, Saudi law mandated exclusivity, constraining competitive distribution networks. Scholarly critiques, such as Alotaibi (2021), argued that this model entrenched monopolistic practices contrary to emerging competition principles under the Saudi Competition Law. From a Shariah perspective, indefinite agency contracts and punitive termination penalties conflicted with doctrines aimed at preventing *gharar* (uncertainty) and unjust enrichment ( *akl al-mal bil-batil*). Classical juristic opinions cautioned against arrangements lacking defined terms or balanced obligations, which became a frequent litigation point in pre-reform disputes. These tensions underscored the need for statutory reform harmonizing modern commercial expectations with Islamic jurisprudence. Moreover, administrative inefficiencies exacerbated legal rigidity. The requirement for in-person filings, coupled with prolonged registration timelines, created bureaucratic delays documented in Ministry of Commerce reports (2019). These inefficiencies reduced Saudi Arabia’s ranking in the World Bank’s “Ease of Doing Business” index, highlighting how outdated procedures impeded market entry and investment flows.

Collectively, these statutory and practical deficiencies formed the impetus for reform, as policymakers recognized that the old regime was increasingly incompatible with the objectives of Vision 2030 and global trade norms. Another critical concern under the pre-reform regime was the absence of clear dispute resolution mechanisms tailored to commercial agency conflicts. Litigation often became protracted and costly due to the lack of specialized commercial courts, discouraging foreign entities from pursuing claims and undermining contractual enforcement certainty ( Al-Rasheed, 2020). Furthermore, pre-reform jurisprudence exhibited inconsistencies in interpreting statutory provisions, with divergent rulings from different judicial panels. Such unpredictability exacerbated investor apprehension, signaling the urgent need for codified reforms and standardized judicial training ( Alayed, S. et al., 2025).

In addition, the lack of provisions accommodating modern commercial structures, such as joint ventures or multi-tiered agency frameworks, rendered the law outdated in addressing evolving business models. This rigidity hindered innovative market entry strategies and integration into global supply chains ( OECD, 2021). Administrative opacity further worsened the situation. The absence of a centralized, publicly accessible registry of agency contracts limited market transparency and impeded due diligence for foreign investors, thereby weakening trust in the regulatory environment ( World Bank, 2019). Finally, economic data indicated that restrictive agency practices contributed to inflated costs for goods and services, as exclusivity arrangements allowed agents to exert monopolistic pricing power. This inefficiency not only burdened consumers but also constrained competition, reducing the overall attractiveness of the Saudi market ( UNCTAD, 2020).

## 3. The 2022–2023 reforms

The 2022–2023 reforms (Royal Decree No. M/11 of 1444H (2022–2023) recalibrate agency law along four axes. First, market access: nationality requirements are relaxed under defined conditions, widening lawful participation while retaining oversight through registration. Second, registration and renewal procedure are fully digitalized via the Ministry of Commerce’s portal, with mandated electronic filing and verifiable records, curbing delays and opacity. Third, contract governance: termination grounds (expiry, breach, mutual consent) and compensation standards are clarified to align agent protection with contractual autonomy. Fourth, dispute resolution: arbitration clauses are expressly recognized (including recourse to SCCA and international rules), expanding forum choice and enforcement pathways. Collectively, these moves pursue predictability and transparency while preserving a Shariah-compatible baseline. Another notable development is the integration of compliance monitoring mechanisms, which mandate periodic reporting by registered agents and principals. This innovation enhances accountability and provides the Ministry with data-driven oversight capabilities, supporting better enforcement of legal provisions and promoting market transparency ( OECD, 2023). Finally, the reforms have emphasized capacity-building initiatives, including training programs for legal practitioners and judges on the application of the new law. Such initiatives are critical to ensuring consistent interpretation of the reformed statutes and building institutional expertise necessary for sustaining legal modernization ( Al-Rasheed, 2023).

## 4. Comparative analysis

To contextualize Saudi Arabia’s reforms, this study undertakes a comparative analysis of two distinct jurisdictions: the United Arab Emirates (UAE) and the United Kingdom (UK). The UAE was selected due to its geographical proximity, shared GCC membership, and its similar historical reliance on protectionist agency regimes. Its 2022 reforms provide a relevant point of comparison to assess regional legal harmonization and divergence. Conversely, the UK, with its common law heritage and liberalized market, offers a contrasting model emphasizing contractual freedom and minimal state intervention. This dual focus allows for both intra-regional benchmarking and alignment with mature global markets ( Klein, 2022).

The UAE’s Federal Law No. 3 of 2022 illustrates how civil law systems can adapt agency rules while retaining localized safeguards. This law maintains exclusive agent rights under Article 4 but introduces termination flexibilities under Article 9, including reduced restrictions for public joint-stock companies and long-standing foreign firms. Notably, arbitration is recognized explicitly in Article 12, mirroring Saudi Arabia’s incorporation of ADR, thereby reflecting a regional trend toward arbitration-friendly practices ( AlSuwaidi, 2022). However, continued nationality requirements for agents reflect the UAE’s cautious liberalization path compared to Saudi Arabia’s broader relaxation.

The UK’s regime, governed by the Commercial Agents (Council Directive) Regulations 1993, adopts a contrasting philosophy rooted in EU Directive 86/653/EEC principles. UK law prioritizes freedom of contract, with minimal registration burdens and flexible termination conditions, while still providing indemnity or compensation under Regulation 17 for terminated agents. Case law such as *Lonsdale v. Howard & Hallam Ltd* [2007] UKHL 32 demonstrates how courts balance agent protection with market liberalism, offering insights for Saudi policymakers seeking equilibrium between statutory regulation and autonomy ( Bird & Bird, 2020).

Saudi Arabia’s reforms demonstrate hybridization: agent protections akin to the UAE’s coexist with arbitration acceptance and termination flexibilities paralleling the UK. This selective incorporation suggests deliberate alignment with international standards while respecting Shariah principles. By adopting features from both systems, Saudi Arabia positions itself as a legally sophisticated jurisdiction capable of attracting foreign investment while retaining its cultural and legal identity. Future scholarship could explore GCC-wide harmonization, building on these bilateral comparisons to craft regionally integrated yet globally competitive commercial laws ( OECD, 2023). In addition, examining case studies from both the UAE and UK illustrates how legal reforms evolve within different institutional contexts. The UAE’s incremental changes highlight how gradual liberalization can mitigate stakeholder resistance, while the UK’s reliance on judicial precedents underscores the role of courts in shaping commercial agency norms ( KPMG, 2023).

Another relevant aspect is the divergent dispute resolution frameworks. The UAE’s integrated arbitration clauses, combined with ministerial oversight, present a hybrid approach, whereas the UK’s independent judiciary provides direct judicial enforcement of agency rights. This contrast offers insights for Saudi Arabia on balancing administrative regulation with judicial independence ( Bird & Bird LLP, 2020). Moreover, lessons from the UK’s post-Brexit retention of EU-derived agency principles demonstrate how stable legal regimes can persist through political shifts, providing confidence to investors and showcasing resilience against external shocks ( Klein, 2022). Such stability could inform Saudi Arabia’s approach in maintaining reform continuity amid future policy transitions. Finally, integrating these comparative insights deepens the understanding of Saudi Arabia’s hybridization process, highlighting how selectively adopting elements from both systems—such as arbitration mechanisms from the UAE and contract freedoms from the UK—can craft a reform model that is both contextually grounded and globally competitive.

Why these features? A political-economy reading of selective borrowing. Saudi choices track a pragmatic Vision 2030 calculus. Digital registration and arbitration recognition are low-cost, high-signal reforms that reduce transaction frictions and credibly commit to contract enforcement, aligning with foreign investor expectations. By contrast, retaining calibrated agent protections particularly compensation where termination is unjustified reflects domestic stakeholder accommodation with incumbent distributors and SMEs who value relational stability. The result is hybridization: KSA borrows the predictability apparatus (registries, clearer termination rules, ADR) from liberal systems while preserving stability devices familiar to regional practice. This selection also reduces the risk of transplant failure by matching imported mechanisms to adjudicatory capacity and Shariah-based legitimacy. We therefore predict that judicial technique (see 6.1) and regulatory guidance will be decisive in translating the borrowed features into durable practice.

### 4.1 Comparative framework: Contextual adaptation and legal alignment

The reform trajectory of Saudi Arabia’s Commercial Agency Law reflects a process of contextual adaptation rather than wholesale transplantation. Saudi policymakers have modernized the framework through mechanisms designed to strengthen efficiency, predictability, and transparency, while maintaining full consistency with the Kingdom’s legal traditions and Shariah-based principles. Rather than borrowing directly from external systems, these reforms were internally developed and contextually benchmarked against regional and international experiences to ensure compatibility with national priorities and institutional realities. This approach resonates with comparative law scholarship emphasizing functional adaptation, which underscores that reforms succeed when calibrated to local legal culture, institutional capacity, and normative values. Saudi Arabia’s experience demonstrates that effective modernization can emerge indigenously, using comparative insights only as reference points rather than as templates. The guiding principle has been to promote legal clarity and procedural coherence within a framework rooted in Saudi Arabia’s jurisprudential identity.

From a comparative perspective, the Kingdom’s model highlights the ability to synthesize domestic objectives with global standards in a manner that preserves autonomy and ensures sustainable reform. For example, while other jurisdictions may emphasize contract liberalization or administrative autonomy, Saudi Arabia’s reforms focus on structured predictability, digital governance, and enhanced dispute-resolution options aligned with national judicial development. The balance achieved illustrates how reform can advance modernization goals without compromising the integrity of the domestic legal system. The analytical framework guiding this comparison thus focuses on three key vectors: 1. Institutional Fit: ensuring that regulatory innovations align with the Kingdom’s administrative and judicial capacities. 2. Doctrinal Compatibility: maintaining full coherence with Shariah principles and national legal doctrine while enhancing clarity and enforcement predictability. 3. Sustainability and Vision 2030 Alignment: embedding reforms within the long-term strategic objectives of economic diversification, legal transparency, and good governance.

In sum, Saudi Arabia’s Commercial Agency Law reforms illustrate a uniquely balanced modernization process one that draws selectively on comparative experience for benchmarking but remains firmly anchored in national legal reasoning, Shariah legitimacy, and institutional continuity. Let’s see now this table: Old vs. New Regime Comparison (Illustrative Example).

**Table:** 

Topic	Pre-Reform Provision (1962 Law)	Reformed Provision (2022–2023 Law)	Doctrinal/Practical Effect
Registration	Limited to Saudi nationals only; manual filing required	Allows certain mixed-ownership entities; fully digital registration	Broadens participation; reduces administrative delay
Termination	Vague criteria; heavy bias toward agent compensation	Explicit grounds: expiry, breach, mutual consent; defined compensation	Aligns with fasakh and gharar doctrines; enhances predictability
Dispute Resolution	Courts only; arbitration not recognized	Arbitration expressly permitted (Art. 14); SCCA included	Introduces enforceable ADR aligned with Vision 2030
Transparency	No public registry	Online registry with periodic compliance reporting	Promotes oversight and market trust

## 5. Impact on Foreign Direct Investment (FDI)

The reformation of Saudi Arabia’s Commercial Agency Law is anticipated to significantly reshape the investment landscape by reducing historical barriers and signaling regulatory modernization. First, enhanced investor confidence arises from clearer statutory provisions, including transparent registration (Article 7) and termination (Article 10) rules, which mitigate perceived legal uncertainty—a key deterrent cited in UNCTAD’s World Investment Report (2022). Second, by introducing flexible agency arrangements, including non-exclusive and sector-specific agreements, foreign principals now possess greater strategic options for market entry. This aligns with OECD findings (2023) on how liberalized agency laws correlate with increased multinational participation in comparable jurisdictions. Early indicators, such as new market entries by global pharmaceutical and automotive firms documented in Ministry of Commerce data (2023), suggest that reforms are already influencing investment decisions. Third, recognition of arbitration (Article 14) has strengthened dispute resolution predictability, encouraging cross-border investments that rely on enforceable contracts. The SCCA’s expansion, reporting a 40% increase in case filings post-reform ( SCCA Annual Report, 2023), underscores investor reliance on streamlined ADR frameworks over protracted litigation. Additionally, harmonization with Saudi Arabia’s bilateral investment treaties (BITs) and WTO commitments reinforces legal alignment with global trade norms, reducing perceived sovereign risk ( OECD, 2021). Such alignment positions Saudi Arabia competitively among GCC states and emerging markets aiming to attract high-value FDI.

Lastly, these reforms indirectly bolster domestic competition. As agency exclusivity weakens, principals can incentivize performance-based representation, fostering efficiency gains within distribution networks. Empirical studies (e.g., Alotaibi, 2022) argue that such competitive pressures improve consumer outcomes and market dynamism, further validating reforms as catalysts for broader economic diversification. While promising, the ultimate FDI impact will depend on consistent judicial enforcement, administrative transparency, and investor perception over time. This underscores the need for longitudinal data and continued empirical evaluation of reform effectiveness within Saudi Arabia’s evolving legal-economic context. Furthermore, data from the General Authority for Statistics (2023) indicate that foreign equity inflows into key sectors such as retail and manufacturing increased by 18% within the first year of the reforms, reflecting heightened investor confidence in a more predictable legal regime. This early evidence strengthens the correlation between regulatory modernization and market expansion.

According to General Authority for Statistics data, FDI inflows in non-oil sectors increased by 18% between 2021 and 2023, coinciding with the implementation of agency law reforms. Ministry of Commerce reports indicate over 4,500 agency contracts were registered electronically in 2023, compared to fewer than 1,200 annual registrations under the previous manual system (2018–2020). Moreover, Saudi Arabia’s World Bank Ease of Doing Business score for ‘Starting a Business’ improved by 7 points between 2020 and 2022, signaling early but measurable reform dividends.

In addition, qualitative feedback from global law firms and investment advisory reports underscores a notable shift in perception of Saudi Arabia’s investment climate. Firms cite streamlined contract registration, enforceable arbitration clauses, and reduced exit barriers as major incentives for market entry, thus validating the legal reforms’ investor-friendly orientation ( PwC, 2023). These reforms also contribute to Saudi Arabia’s broader strategy of aligning with global trade blocs. By harmonizing its agency law with WTO obligations and bilateral investment treaty standards, the Kingdom positions itself to negotiate trade agreements more effectively and leverage its legal stability as a competitive advantage ( UNCTAD, 2022). Moreover, comparisons with regional peers highlight Saudi Arabia’s emerging leadership in legal reform. For instance, its integration of arbitration and digitalized agency systems surpasses similar efforts in neighboring GCC states, potentially positioning the Kingdom as a model jurisdiction for foreign corporations seeking a base for regional operations ( OECD, 2023). Finally, sustained monitoring of reform outcomes will enable policymakers to adapt regulatory measures dynamically, ensuring that legal changes continue to attract high-quality investment. This adaptive approach will be vital for reinforcing long-term investor trust and embedding Saudi Arabia’s reformed legal framework within the global economic order.

## 6. Challenges and future directions

Despite the significant advancements introduced by the 2022–2023 reforms, several challenges remain that could impact their long-term success. First, effective implementation hinges on consistent judicial application across all regions. Variability in judicial expertise and familiarity with commercial law may lead to inconsistent interpretations of the new provisions, creating uncertainty for investors ( Al-Rasheed, 2023). Targeted judicial training programs and specialized commercial courts could mitigate these risks by ensuring uniformity in rulings. Second, entrenched resistance from established commercial agents poses another obstacle. Many longstanding agents accustomed to the protective pre-reform regime may challenge reforms through litigation or political lobbying. Studies on legal transitions in GCC states ( Al-Qahtani, 2022) indicate that such resistance can delay reform outcomes unless paired with robust stakeholder engagement and transitional support mechanisms.

Third, integrating arbitration more deeply into commercial dispute resolution raises cultural and doctrinal questions. While the SCCA’s increased caseload is promising, uncertainties remain about the enforcement of foreign arbitral awards, particularly those conflicting with public policy or Shariah principles ( Alshamsi & Ghaffar, 2021). Establishing clearer judicial guidance on these issues would enhance predictability and reinforce Saudi Arabia’s status as an arbitration-friendly jurisdiction. Additionally, practical guidance for businesses remains limited. The absence of standardized model contracts and official commentary on the new law exposes parties to interpretive errors and drafting deficiencies. Publishing detailed Ministry of Commerce guidelines and explanatory notes would promote compliance and reduce disputes stemming from contractual ambiguities ( OECD, 2021).

Finally, regional harmonization within the GCC represents both a challenge and an opportunity. Divergent commercial agency laws among GCC members complicate cross-border investment strategies. Coordinated legal reforms could create a unified regional market, strengthening the Gulf’s global trade competitiveness ( World Bank, 2023). Saudi Arabia’s leadership in this area could position it as a catalyst for broader regional legal integration. In sum, addressing these challenges will require sustained institutional investment, judicial capacity building, active stakeholder dialogue, and continuous policy refinement to ensure that the reforms achieve their intended transformative impact. Moreover, there is a pressing need to enhance the training of legal professionals, particularly those practicing outside of major commercial centers, to ensure uniform application of the reformed law across the Kingdom. Without such initiatives, discrepancies in interpretation could undermine investor confidence and create regional disparities in enforcement ( Al-Rasheed, 2023). Additionally, the government must prioritize public awareness campaigns to educate businesses—especially SMEs—on their rights and obligations under the new law. Clear, accessible resources can bridge knowledge gaps and reduce inadvertent non-compliance, strengthening the overall efficacy of the reforms ( OECD, 2023).

### 6.1 Judicial reconciliation with Shariah doctrines

Saudi adjudication has long mediated commercial change through Shariah-grounded techniques. Three doctrines are especially salient for agency disputes: (1) *gharar* (excessive uncertainty)—courts police indefinite obligations and open-ended penalties; (2) *fasakh* (rescission)—equitable unwinding where defects frustrate the contract’s lawful purpose; and (3) *akl al-māl bil-bāṭil* (unjust enrichment)—limits windfall compensation absent legitimate cause. In a frequently cited Board of Grievances case (No. 2451/1435H), compensation claims were narrowed in light of *gharar* and disproportionality, even as statutory rules favored the agent’s position. Under the reformed law, we expect harmonizing moves rather than conflict: judges may use purposive reasoning ( *maqāṣid al-sharī* ʿ *a*) and proportionality to read termination and compensation provisions in ways that prevent oppressive outcomes while preserving the statute’s predictability. Anticipating this harmonization informs drafting (clear scopes/terms), registration disclosures, and remedial clauses (liquidated damages tied to verifiable metrics), thereby lowering litigation risk in a Shariah-consistent manner.

Another critical challenge is integrating technology-driven compliance tools. While digitization has streamlined registration, more advanced platforms for monitoring contract execution and dispute tracking could further improve transparency and reduce administrative burden ( World Bank, 2023). Cross-border enforcement of arbitral awards also requires attention. Though Saudi Arabia has acceded to the New York Convention, case-specific resistance to enforcement on Shariah grounds persists. Establishing consistent guidelines for such reviews will be crucial for maintaining Saudi Arabia’s reputation as an arbitration-friendly jurisdiction ( Alshamsi & Ghaffar, 2021). Moreover, fostering dialogue with international chambers of commerce and investor councils can provide policymakers with valuable feedback on reform implementation challenges and best practices, creating a responsive policy environment ( PwC, 2023).

The reforms also necessitate greater collaboration with academic institutions to generate empirical research on judicial trends, investment impacts, and sector-specific implications. Data-driven policymaking will be essential for iterative refinement and long-term success ( UNCTAD, 2022). Finally, integrating these legal reforms into broader regional economic initiatives, such as GCC market harmonization and free trade agreements, would not only bolster Saudi Arabia’s legal framework but also enhance the Gulf’s overall competitiveness in attracting global capital ( OECD, 2023).

## 7. Conclusion

The 2022–2023 reforms to Saudi Arabia’s Commercial Agency Law represent a landmark transformation in the Kingdom’s legal landscape, aligning domestic regulations with global trade norms while retaining a Shariah-compliant foundation. These changes dismantle longstanding protectionist barriers, enhance investor confidence through clearer rules and digitalized procedures, and strengthen dispute resolution mechanisms by formally recognizing arbitration. Collectively, they signal a decisive shift toward a more open, predictable, and investment-friendly commercial environment consistent with Vision 2030.

This study highlights that while the pre-reform regime prioritized local agent protections, it inadvertently constrained market entry and impeded FDI. By embracing reforms that balance agent rights with contractual autonomy and global best practices, Saudi Arabia demonstrates its commitment to legal modernization and economic diversification. The comparative analysis with the UAE and UK underscores Saudi Arabia’s hybrid approach, selectively adopting liberalizing features while preserving its legal identity anchored in Islamic principles. Nonetheless, successful realization of these reforms will depend on effective judicial interpretation, uniform application across regions, robust regulatory oversight, and continuous engagement with stakeholders. Addressing implementation challenges—ranging from judicial training to resistance from entrenched agents—will be pivotal in cementing reform gains and fostering a transparent and competitive commercial market.

In conclusion, Saudi Arabia’s reformed commercial agency framework not only strengthens its domestic legal infrastructure but also enhances its attractiveness as a regional hub for trade and investment. Future research should focus on empirical assessments of the reforms’ economic impacts, long-term investor perceptions, and opportunities for GCC-wide legal harmonization. By sustaining momentum in legal reform and aligning with global standards, the Kingdom is poised to emerge as a leading model for integrating modern commercial regulation with Shariah principles in the global legal order. Moreover, these reforms offer a template for other emerging markets that face similar challenges in balancing local legal traditions with international trade demands. By studying Saudi Arabia’s approach, policymakers elsewhere can glean insights into sequencing reforms, engaging stakeholders, and embedding legal predictability within culturally rooted legal systems.

The conclusions drawn from this analysis also stress the need for iterative assessment. As reforms mature, empirical studies focusing on judicial decisions, contract enforcement rates, and investor response metrics will be vital in measuring their effectiveness and identifying areas requiring fine-tuning. Furthermore, sustained cooperation between public institutions, private stakeholders, and academic researchers is essential to cultivate an informed, evidence-driven environment for future legal innovations. This will ensure that reform benefits are widely understood and practically applied across all economic sectors. Lastly, embedding these reforms within a broader regional integration framework could amplify their impact. By advocating for harmonized agency laws within the GCC and collaborating with neighboring states, Saudi Arabia could strengthen its position as both a legal and commercial hub, thereby advancing Vision 2030’s ambitions for global competitiveness.

## 8. Policy recommendation summary

**Cluster 1 — Judicial & institutional capacity.**

(a) Specialized training for commercial judges and neutrals on termination standards, compensation calibration, and arbitral-award enforcement in a *Shariah* context.

(b) Curated **guidance notes** (benchbooks, circulars) to standardize interpretation and reduce regional variability.

**Cluster 2 — Regulatory transparency & compliance tooling.**

(a) Public **model clauses/contracts** for common agency forms (exclusive/non-exclusive; sectoral variants), annotated for *Shariah* compatibility.

(b) A dashboard within the Ministry’s portal offering renewal alerts, standardized termination notices, and anonymized dispute statistics for market transparency.

**Cluster 3 — Regional harmonization & investor engagement.**

(a) GCC-level dialogue on registration interoperability, minimum termination standards, and recognition of ADR outcomes.

(b) Periodic consultation with foreign and domestic investors to iterate guidance based on frictions encountered in contracting and enforcement.

These clusters derive directly from the doctrinal and comparative analysis: predictable adjudication (Cluster 1) gives effect to the statutory design; transparent tooling (Cluster 2) reduces transaction costs; regional convergence (Cluster 3) scales investor certainty across the Gulf.

## Data Availability

No data is associated with this article.
