# Modernizing Commercial Agency Regulations in Saudi Arabia: Legal Reforms and Comparative Insights

**DOI:** 10.12688/f1000research.168970.4

**Published:** 2026-01-21

**Authors:** Abdullah Ali Alasmari, Hajed A. Alotaibi

**Affiliations:** 1 Assistant Professor, Department of law, College of judicial studies and regulations, Umm Alqura University, Mecca, Mecca, Saudi Arabia; 2 Associate Professor, Department of Sharia, College of Sharia and Law, Majmaah University, Al Majmaah, Riyadh Province, 11952, Saudi Arabia

**Keywords:** Saudi Arabia, Commercial Agency Law, Legal Reform, Shariah Compliance, Arbitration, Foreign Direct Investment, Comparative Law, Vision 2030.

## Abstract

**Background:**

This study analyzes Saudi Arabia’s 2022–2023 reforms to the Commercial Agency Law through legal, economic, and comparative lenses. The pre-reform regime—marked by rigid nationality limits, procedural burdens, and litigation-prone termination rules—constrained market entry and investor confidence.

**Methods:**

Using a doctrinal approach to statutes and implementing regulations, triangulated with Saudi judicial practice and policy instruments, we benchmark Saudi reforms against the UAE and UK. A Shariah lens clarifies how *gharar* (uncertainty), *fasakh* (rescission), and unjust enrichment ( *akl al-māl bil-bāṭil*) shape agency disputes.

**Results:**

Key changes include more flexible nationality rules, end-to-end digital registration and renewal, clearer termination/compensation standards, and formal recognition of arbitration (including SCCA). Together these measures enhance contractual predictability and lower compliance frictions, with early indications of improved market transparency and investor sentiment.

**Conclusions:**

The reforms signal a hybrid model—liberalization aligned with global practice while preserving a Shariah-grounded identity. Remaining priorities include consistent judicial application, practitioner training, clearer guidance, and GCC coordination. We propose thematically grouped policy steps to consolidate gains and support Vision 2030’s diversification agenda.

## 1. Introduction

Over the past few years, the Kingdom of Saudi Arabia (KSA) has experienced several economic and legal reforms as part of the ambitious Vision 2030. Among the key areas of regulation influenced by these changes is commercial agency law, which underpins the formation of business relationships between local agents and overseas principals. Traditionally, the Saudi commercial agency laws were initially defined by Royal Decree No. M/11 of 1382H (1962) was marked with the protectionist policy and strong restrictions on foreign principals. Although these rules were meant to empower local businesses and protect national economic interests, they tended to yield inflexible business regulations that discouraged foreign direct investment (FDI) and market liberalization.

There is an increasing scholarly literature explaining why commercial agency regimes need to be modernised to strike the right balance between investor security and market competitiveness. Regarding the self-inflexibility of pre-reform systems, restrictive agency laws increase transactional risks and reduce the entry of multinational corporations into regional markets. In the same way, comparative law analysis of jurisdictions like the UAE and the UK shows that the presence of predictability in the law, a well-developed and accessible dispute resolution system, and concise compensation guidelines leads to a high level of investor confidence ( Sornarajah, 2021). All these works aim to address a research gap. As discussions of the reform increase, little research offers a closer doctrinal and comparative examination of recent agency reforms in Saudi Arabia and how they fit within both Shariah and global best practices.

Furthermore, some researchers, including Alotaibi (2021), have noted the overlap between Shariah jurisprudence and current commercial law, arguing for legal paradigms that can merge Islamic principles with contemporary economic conditions. For example, the gharar (uncertainty) principle traditionally complicated the contract of open-ended agency. Still, recent reforms have sought to bring these principles into international market standards ( El-Gamal, 2006). This two-sidedness offers fertile ground for legal investigations, particularly in assessing how Saudi Arabia can remain an Islamic legal entity while also incorporating market-driven changes compatible with international trade regimes.

Furthermore, the adoption of these reforms overlaps with Saudi Arabia’s obligations under international conventions, such as WTO membership and bilateral investment treaties, and it is crucial to consider how these reforms will fit within international trade regulations ( Sornarajah, 2021). The discussion of these dimensions helps clarify how Saudi Arabia positions itself in the global commercial realm. The new Commercial Agency Law meets these requirements by outlining procedures for registration, termination, and dispute resolution. It minimizes legal ambiguity without reducing the Kingdom’s core regulatory freedom. This situational connection makes the reforms internationally relevant without exaggerating their importance within a broader law-and-development discourse.

Even the application of Shariah-based principles in contemporary legal systems raises further essential questions about judges’ interpretation and application of the laws. The study illuminates how Islamic jurisprudence has been modified in response to the commercialization of global economies by examining how the courts strike a balance between religious beliefs and modern commercial standards ( Hallaq, 2009). Furthermore, the study can be included in discussions of hybrid legal systems and of Saudi Arabia’s role in providing its population with aspects of civil law, common law, and Islamic legal traditions ( Otto, 2010). This hybridity offers a new avenue for other jurisdictions seeking to modernize while retaining cultural and religious identity.

The main contribution of this article is the development of a three-vector model of institutional fit, doctrinal compatibility, and alignment with Vision 2030 to assess commercial agency reforms in a Shariah-based legal system. Based on this framework, the paper demonstrates that recalibrating agency law-investment policy through it creates a unique hybrid model that cannot be diluted into either regional practice, as seen in the UAE, or the UK’s liberal agency regime. By doing so, it transcends traditional legal transplant scripts by showing how reforms in Saudi Arabia incorporate international investor-friendly characteristics into a normative framework grounded in Shariah and domestic institutional constraints. In cases where this research draws on policy reports, they can only be used as contextual pointers to the modern-day reform processes. The article’s analytical premises are primarily based on academic sources in comparative law, commercial jurisprudence, and Islamic commercial law, as well as on the institutional adaptation theory, which serves as the framework for interpreting the statutory changes ( Reimann & Zimmermann, 2019).

**Conceptual Framing:** The discriminatory integration of foreign principles into Saudi Arabia’s commercial agency law invites the traditional discourse on legal transplant and institutional adjustment. Modernization can be enhanced by transplant, but borrowed rules cannot work without being fitted to local organizations and normative anchors ( Reimann & Zimmermann, 2019). In a mixed system grounded in the Shariah, the risk is not formal incompatibility but interpretive friction, unless adjudicative practice and administrative guidance develop concomitantly ( Badr, 1978). This also places the reforms within the law-and-development discourse, which balances the standard-setting pressures of globalization with legal particularism and legitimacy ( Cane & Kritzer, 2010).

In this paper, we consider the post-2022 regime in Saudi Arabia as a strategy of hybridization: we are liberalizing, specifically in the areas of digitalization, adjudication, and clarity in termination, while applying agent safeguards in moderation to maintain the stability of relations. We test two implications in the paper: (i) does hybridization enhance predictability among foreign principals without impairing the coherence of doctrine among domestic actors; (ii) is the success of transplanted features mediated by institutional adaptation (judicial technique, regulatory guidance, and practitioner capacity) ( Sfeir, 2007).

The strategic context of Vision 2030 increases the significance of this question. Vision 2030 expressly aims to diversify the Saudi economy away from oil reliance, encourage investment, and attract international capital into sectors beyond petroleum ( Ramady, 2010). Legal modernization is a cornerstone of this change and signals to global investors that Saudi Arabia is determined to establish a transparent, predictable, and enforceable regulatory environment. The commercial agency reforms can therefore be interpreted not just as technical adjustments to the legal framework but also as a tactical tool used to achieve broader economic goals, such as rising FDI inflows, competitiveness, and internationalization ( O’Kane, 2013).

Moreover, the reforms are in line with Saudi Arabia’s current commitments under WTO regulations and bilateral investment agreements, which set out the conditions for market access and the non-discriminatory treatment of foreign investors ( Sornarajah, 2021). The updated Commercial Agency Law minimizes legal uncertainty by clarifying the registration, termination, and dispute-resolution mechanisms; however, it does not diminish the Kingdom’s regulatory discretion. This is the two-fold success of the following: modernization without loss of sovereignty; a complex balancing act that deserves the close attention of scholars ( Ballantyne, 1986).

The other aspect of the conceptual framing concerns the roles of legal culture and judicial capacity as mediators of reform outcomes. That even well-crafted laws cannot always have the desired impact unless the court system is up to speed on the new legislative provisions, the administrative agencies are receptive to change, or business sectors are not convinced that the law is closely followed ( Foster, 2010). Therefore, the improvements in agency law reforms in Saudi Arabia are not solely reliant on the quality of statutory texts but also require complementing investments in judicial education, regulatory advice, and stakeholder involvement. This is where the role of an institutional lens that looks not only at what the law says but how it is being interpreted, applied, and experienced in practice becomes important ( Cane & Kritzer, 2010).

### 1.1 Methodology and sources

This paper takes the form of a doctrinal legal research approach, which involves a systematic examination of primary legal texts, including Royal Decree No. M/11 of 1444H (2022-2023), which establishes regulations provided by the Ministry of Commerce and the provisions of some of its laws. The doctrinal method offers an opportunity to thoroughly explore the statutory text, its interpretation by the Saudi courts, and its alignment with accepted principles of commercial law ( Reimann & Zimmermann, 2019). Comparative analysis is used to supplement the research, focusing on the UAE as a regional counterpart with a civil law system and the UK as an ordinary law jurisdiction with historically liberalized commercial systems. This two-fold comparison offers both regional and global benchmarking, enabling comparisons of Saudi reforms with legal traditions and regulatory philosophies from other regions ( Beraudo, 2014).

Moreover, the paper combines a Shariah-grounded analytical approach, which explores the impacts and interactions of Islamic jurisprudence on contemporary commercial regulation. This includes an evaluation of the academic literature on principles such as gharar and fasakh, their use in agency-related litigations, and their application in Saudi judicial practice ( Nyazee, 1998). Lastly, the methodology also involves a secondary literature review, comprising peer-reviewed journal articles, practitioner comments, policy reports by agencies, including UNCTAD and OECD, and data from investment and arbitration centers. This three-pronged strategy will ensure that the research does not merely provide an interpretation of the legal texts but also places them in the broader economic, judicial, and policy frameworks ( Cane & Kritzer, 2010).

To address the doctrinal and comparative analysis, we rely specifically on the theoretical perspectives presented in the Introduction. We read why particular characteristics (e.g., arbitration recognition, digital filing, compensation calibration) were chosen and how they will probably engage in a Shariah-grounded adjudicative culture through legal transplant and institutional-adaptation perspectives ( Reimann & Zimmermann, 2019). This does not engage in only descriptive comparisons, but also establishes our empirical baselines in the FDI and registration arenas. The corpus of legal texts considered will cover the period 2018-2024 to ensure methodological transparency in the analyzed content: both pre-reform and post-reform practice are represented. Inclusion criteria included any circulars or ministerial directives publicly available on the Ministry of Commerce’s digital portal, as well as secondary literature that provides interpretative background. The exclusion criteria included unpublished or inaccessible administrative guidance.

The doctrinal analysis is done at the level of the clauses, and where necessary, where the operative statutory text is quoted and interpreted in relation to the Shariah maxims (qawaa id fiqhiyyah) and comparative statutory analogies. It is specially noted how modern statutory provisions have been reconciled with the Shariah concepts of gharar (uncertainty), fasakh (rescission), akl al-māl bil-bait (unjust enrichment) by the Saudi courts ( Hallaq, 2009). The comparative aspect examines purposive sampling under the UAE Commercial Agency Law (Federal Law No. 3 of 2022) and the UK Commercial Agents (Council Directive) Regulations 1993. The comparative assessment is based on a three-step rationale: (1) to determine the statutory parallels, (2) to analyze how it fits within local institutional constraints, and (3) to examine cross-system transferability ( Beraudo, 2014). The theoretical framing is based on legal transplant and institutional adaptation theories, which explain how specific reforms were selectively borrowed and how they interact with the culture of adjudication under Shariah in Saudi Arabia. This enables the paper to go past description to explain how and why specific characteristics travel and succeed ( Reimann & Zimmermann, 2019).

This same order of UAE and UK subsections is thus provided: market access and registration, termination and compensation, and dispute-resolution pathways, to allow a functional comparison between them. Throughout the paper, there has been a clear distinction between the two types of sources. The primary documents consulted are policy and practitioner documents (OECD, UNCTAD, law-firm reports, etc.), which are used to show recent practice, the administration’s trends, and the market’s perception of reforms. In comparison, the conceptual framing, doctrinal critique development, and the placement of the Saudi Arabian reform agenda within existing academic discourses are developed using scholarly sources, such as works on legal transplants, comparative legal practice, and Shariah-commercial jurisprudence ( Foster, 2010).

The rigor of the method ensures that the study’s findings are based on verifiable facts, theoretically informed, and practically relevant. The integration of accuracy into the doctrine, combined with comparative and Shariah analyses, makes the research a comprehensive assessment of commercial agency reforms in Saudi Arabia, which is both scholarly and policy-based ( Cane & Kritzer, 2010).

### 1.2 Objectives

This research has fourfold objectives. First, it attempts to determine the organizational flaws of the Saudi Arabia pre-reform regime of commercial agency, particularly its statutory and institutional rigidity, its inefficiency, and its discouraging impact on investment. Second, it will examine the legal and Shariah-compliant characteristics of the 2022-2023 reforms, with a specific focus on the newly introduced registration, termination, and dispute-resolution provisions. Third, it draws a comparison between the reformed agency law of Saudi Arabia and the Federal Law No. 3 of 2022 in the UAE and the Commercial Agents (Council Directive) Regulations 1993 in the UK, which offers an insight into similarities, differences, and lessons of harmonization of laws on agency ( Beraudo, 2014). Lastly, it measures the impact of such reforms on FDI and legal modernization and provides evidence-based recommendations to policymakers ( Sornarajah, 2021).

In addition to these primary objectives, the research also aims to contribute to the theoretical literature on reconciling Shariah-based principles with worldwide systems of commercial law ( Vogel & Hayes, 1998). In that way, it offers a delicate perspective on the study of legal reform in Islamic jurisdictions where cultural, religious, and economic goals converge. The study will also seek to educate policymakers, legal practitioners, and academicians on best practices for applying law reform in emerging markets ( Otto, 2010). It helps fill the gap between theoretical notions and actual practice by dissecting statutory provisions and judicial practices, and offering practical advice on modernizing the law ( Cane & Kritzer, 2010).

Finally, the research aims to lay the groundwork for future empirical studies by identifying quantifiable measures, including dispute resolution, agency registrations, and investment flows, that can determine the continued effectiveness of these reforms. This will serve as a long-term evaluation roadmap to ensure that legal development aligns with both the Vision 2030 goals and global market expectations ( Ramady, 2010). Although the research engages with economic indicators and policy implications, these factors serve as supporting background rather than equal pillars in the analysis. The fundamental area of the study is a doctrinal and comparative study of the 2022-2023 Commercial Agency Law reforms, its statutory interpretation, Shariah coherence, and systematic cross-jurisdictional benchmarking ( Reimann & Zimmermann, 2019).

An important task is also to examine how the reforms strike a balance between conflicting interests. On the one hand, they have to safeguard the legitimate expectations and investments of the local agents who have been under the previous regime for decades ( Ballantyne, 1986). Conversely, they have to establish adequate flexibility and predictability to be appealing to foreign principals who require modern, transparent, and enforceable contracts ( Sornarajah, 2021). This balancing surgery is both a technical and legal issue and a socio-economic and political issue because it involves balancing domestic stakeholder interests and international market expectations ( O’Kane, 2013).

In addition, the paper seeks to examine how digitalization can be applied to legal reform. The transformation of the manual registration system to fully digital platforms is a radical shift in the formalization and control of commercial relationships. The key to assessing the success of the overall reforms is understanding the implications of such a shift for administrative efficiency and transparency ( Ramady, 2010).

Lastly, the research identifies potential areas for future reform. Although the amendments of 2022–2023 may be considered a significant step, there is no perfect or complete legal system. The research can be used in a continuous process of legal refinement and enhancement by establishing prevailing gaps, ambiguities, or areas of dispute ( Foster, 2010). This is a forward-looking approach, making the study not only retrospective but also proactive, and providing guidance on the next steps in the legal development process in Saudi Arabia.

## 2. Pre-reform legal framework

Prior to the recent reforms, the commercial agency regulation in Saudi Arabia was mainly controlled by the Commercial Agency Law, which was passed by royal decree No. M/11 of 1382H (1962). This system was characteristic of the protectionist economic direction of the time and had extreme demands of localization. Commercial agents were to be solely Saudi nationals, or wholly owned by a local registered company registered with the Ministry of Commerce. Contracts signed with unregistered agents were not binding, and no rights were enforceable by the foreign principals ( Ballantyne, 1986). Although this regime was meant to protect local commercial interests, it indirectly affected foreign investment, raising concerns about whether it would be enforced and whether it would be legally certain ( O’Kane, 2013).

The pre-reform regime was accused of one of its most significant failings: granting excessive protection to agents, especially in contract dissolution and compensation. The statutory provisions, which prioritized the protection of agents while disregarding the autonomy of the contract, led courts to award compensation to agents in cases where term-based contracts had expired. As an illustration, judicial practice before the Board of Grievances showed a tendency to prioritize statutory protection over commercial principles, making the compensation process more uncertain and risky to litigate on behalf of foreign principals ( Alzahrani, 2024). Academic commentators have noted that these interpretative trends are creating a sense of legal uncertainty rather than expressing a unified jurisprudential doctrine ( Alayed et al., n.d.).

The law’s inflexibility also inhibited flexibility in international business practices. The exclusivity was obligatory, and non-exclusive, regional, or sector-specific agency agreements were not allowed, as are generally employed in comparative jurisdictions ( Beraudo, 2014). This framework gave rise to monopolistic behaviour and was incompatible with the new competition standards set out in Saudi competition policy ( Alotaibi, 2021). The indefinite agency structure and punitive termination results were also questionable in terms of gharar (undue uncertainty) and unjust enrichment (akl al-m2al bilb2til), both of which are undesirable in Islamic commercial jurisprudence ( El-Gamal, 2006; Nyazee, 1998).

Administrative inefficiencies exacerbated these shortcomings in the law. The registration processes were primarily submitted physically, and had long processing times with very little transparency ( Ramady, 2010). This was further impaired by the lack of a centralized, publicly accessible registry of agency agreements, which impeded due diligence by foreign principals and weakened market transparency ( O’Kane, 2013). Such bureaucratic hurdles were the cause of Saudi Arabia’s deteriorating performance in global ease-of-doing-business indices and of limitations on gaining access to the market.

The pre-reform method of dispute resolution was neither transparent nor efficient. Arbitration was not explicitly provided for in the law to apply to agency disputes. However, Saudi Arabia had ratified the New York Convention and even established arbitration institutions, including the Saudi Center of Commercial Arbitration. This confusion raised questions about the enforceability of arbitration provisions and deterred foreign entities from seeking alternative dispute resolution methods ( Saleh, 1984; Al-Ramahi, 2008). Inconsistency among courts in interpreting statutory provisions also heightened investors’ anxiety ( Alayed et al., n.d.).

Exclusive agency practices were also economically associated with less competition and higher consumer prices because agents held monopolistic pricing power ( Ramady, 2010). These results were not in line with the Kingdom’s overall objectives to diversify its economy, attract foreign direct investment, and participate in global value chains ( Sornarajah, 2021). As Saudi Arabia’s economy became more mature, the protectionist rationale that underpinned the 1962 law increasingly conflicted with modern commercial realities, and the need to reform the legislation on a large scale became evident. The following reforms were aimed at addressing these structural deficiencies and harmonizing the statutory rules with the principles of the Shariah and the latest commercial practices.

## 3. The 2022–2023 reforms

These are the 2022-2023 reforms introduced by Royal Decree No. M/11 of 1444H (2022-2023) and recalibrate the agency law on four principal axes. To begin with, access to markets: nationality conditions are liberalized, subject to specified conditions that expand the scope of legal participation, but control is maintained through registration ( O’Kane, 2013). One of the most probable aspects of interpretative ambiguity is the extent to which mixed-ownership participation is permissible and whether the indirect foreign ownership structure would fall within the scope of the reform. The courts can ask such questions in a purposeful manner that encourages an economic element and regulatory purpose, especially where Shariah-based aspects of justice and openness are involved ( Hallaq, 2009).

Second, registration and renewal processes are entirely computerized through the Ministry of Commerce portal, which requires electronic filing and verifiable records, thereby reducing delays and the extent of obscurantism ( Ramady, 2010). With electronically submitted documents records being the new norm, there can be conflict over the weight of evidence for such records and the legality of digitally authenticated documents. The judicial committees will therefore take into account the emerging e-transactions jurisprudence and administrative circulars to standardize admissibility and eliminate procedural uncertainty ( Foster, 2010).

Third, control over contracts: grounds of termination (expiry, breach, mutual consent) and compensation norms are made explicit in a way that does not conflict with contractual autonomy and agent protection. The issues of what constitutes justified termination or commensurate compensation may fall into a grey area, particularly in long-term commercial relationships. These provisions are to be interpreted by Saudi courts through the application of proportionality analysis and Shariah teachings, such as gharar and fasakh, to ensure that compensation remains linked to verifiable harm rather than an automatic entitlement ( El-Gamal, 2006; Nyazee, 1998).

Fourth, dispute resolution: dispute resolution approaches are explicitly acknowledged (such as resorting to the SCCA and international rules), thereby expanding both the scope of forum selection and the enforcement options ( Saleh, 1984). Concerns about the scope of arbitrability can also arise, as can issues regarding the application and enforcement of foreign arbitral awards in instances involving public policy or Shariah issues. Cases The trend of recent years towards aligning domestic law with international norms is also expected to be reflected in judicial interpretation, without forfeiting fundamental doctrinal restraints on unconscionable or excessively uncertain contractual terms ( Al-Ramahi, 2008; Yasin, 2012).

All these actions are basically aimed at seeking predictability and transparency without altering a Shariah-compatible floor ( Hallaq, 2009). The integration of compliance monitoring mechanisms is another interesting development that requires periodic reporting by registered agents and principals. This invention increases accountability and provides the Ministry with data-driven supervisory features that will improve the implementation of the law and foster a transparent market ( Ramady, 2010).

Lastly, the reforms have also focused on capacity-building measures, such as training legal practitioners and judges on the implementation of the new law ( Foster, 2010). These programs are essential to maintaining uniform interpretation of the reformed statutes and to building institutional competence required to sustain legal modernization. The computerization of the registration processes is one of the most important changes. The new system requires that all contracts between agencies be registered electronically through the Ministry of Commerce’s online portal. It is a system that enables real-time monitoring of application status, automated verification of documentation, and safe storage of contracts in a central database ( O’Kane, 2013). Search is also made possible through the digital platform, where interested parties can determine whether agency agreements exist for specific products or market segments, thereby reducing information asymmetry that creates due diligence challenges ( Ramady, 2010).

Another significant change is the elucidation of the grounds of termination. The restructured legislation explicitly provides for justifiable grounds for termination, comprising contract expiry, material breach, mutual consent, and force majeure. Such codification reduces the discretion of judicial bodies and provides contracting parties with more precise instructions regarding their rights and duties. Furthermore, under the law, proportionality is applied to compensation claims: compensation can be proportional only to actual damages incurred and cannot be punitive in the absence of egregious misconduct ( Beraudo, 2014).

The establishment of arbitration as an acceptable dispute-resolution tool marks a paradigm shift. The reformed law in Article 14 expressly allows parties to incorporate arbitration clauses into their agency agreement and to accept the jurisdiction of the SCCA and other endorsed arbitral institutions ( Saleh, 1984). This clause complies with international commercial practice, and the Kingdom shows interest in offering a variety of effective methods for resolving disputes ( Al-Ramahi, 2008). The express indication of arbitration also covers past issues of enforceability, thereby minimizing legal ambiguities and encouraging foreign principals to invest more confidently in the Saudi market ( Yasin, 2012).

The loosening of nationality requirements, albeit with certain limitations, introduces new opportunities for the foreign specifications of agency relations ( Sornarajah, 2021). Under the new legislation, there are now categories of foreign-owned entities that can become registered agents, provided they meet the outlined requirements regarding capital investment, operational capacity, and regulatory compliance. This selective liberalization widens the range of possible agents, increases competition, and offers foreign principals greater choices about how to enter the market and distribute ( O’Kane, 2013).

However, the reforms are well-balanced, carrying out liberalization while preserving genuine agent interests. The law still contains clauses requiring good-faith negotiation, fair dealing, and reasonable notice of termination. It also maintains agents’ rights to compensation in cases of termination other than for failing to act in good faith or for actions by the principals that diminish the agency relationship. This balance represents a high level of knowledge of the necessity of impressing foreign investors, as well as of preserving social and economic stability by securing old business relations ( Vogel & Hayes, 1998).

The compliance monitoring mechanisms are incorporated, and this element of regulatory control has added another dimension. Registered agents and principals are now required to file regular reports on their commercial operations, sales volumes, experience in dispute resolution, and any significant alterations to the agency relationship ( Ramady, 2010). This information helps the Ministry of Commerce track market dynamics, identify trends in non-adherence, and take proactive measures to eliminate potential conflicts or anti-competitive behavior. These reporting requirements also benefit market participants by increasing the amount of available information about the business environment ( Foster, 2010).

Capacity-building programs are an addition to the statutory reforms that target institutional issues in implementation. With the support of legal and commercial institutions, the Ministry of Commerce has initiated training on the implementation and interpretation of the new law for judges, arbitrators, lawyers, and business individuals ( Cane & Kritzer, 2010). These courses include digital evidence, Shariah-compliant contract interpretation, international arbitration, and comparative commercial law. The reforms also aim to ensure that the legal infrastructure is well-positioned to support the new statutory framework by investing in human capital development ( Foster, 2010).

The reformed law also includes transitory provisions to address the transition from the old regime to the new one. The current agency contracts registered under the old law remain valid, provided they comply with the latest reporting and registration requirements within the specified time frames. Contract parties are urged to renegotiate under the new law to comply with its provisions. However, the legacy provisions can still be enforced in areas where they do not interfere with the compulsory regulations in the new law. This intermediate solution aims to reduce the impact and promote a gradual pace of adaptation to the latest legal norms ( Beraudo, 2014).

## 4. Comparative analysis

In an attempt to put Saudi Arabia’s reforms into context, this paper will conduct a comparative study of two jurisdictions: the United Arab Emirates (UAE) and the United Kingdom (UK). The UAE was chosen based on its geographic proximity, its status as a GCC member, and its comparable past dependence on protectionist agency regimes. Its 2022 reforms provide an appropriate basis for evaluating regional legal harmonization and divergence. In contrast, the UK, with a common law tradition and a liberalized market, provides a different example that emphasizes the freedom of contract and a low role for the state. This two-way focus enables one to conduct intra-regional benchmarking and align with the global market ( Klein, 2022).

This comparative analysis is systematic, utilising a three-dimensional framework to ensure methodological clarity and functional equivalence across jurisdictions. First, we examine how the registration and market-access rules are handled in a regulatory context, as they establish the legal bar to entry for an agency. Second, we compare termination and compensation systems regarding their relative merits in providing contractual freedom and safeguarding the agent. Third, we evaluate the design of dispute resolution, especially the accessibility and enforceability of arbitration compared with court-based options. The comparison, organised around these three vectors, would enable the similarities and differences between the UAE and UK regimes to be evaluated systematically and directly in relation to the characteristics of the Saudi reforms.

The Federal Law No. 3 of 2022 in the UAE is an example of how the civil law system can be flexible, enabling agency regulations to adapt without abandoning localized protection. Sharing similarities with Article 4, but also providing flexibilities in termination under Article 9, such as minimum restrictions on long-established foreign corporations and single-stock companies, this law retains the exclusive rights of agents. It is interesting to note that Article 12 expressly mentions arbitration, reflecting the adoption of ADR in Saudi Arabia and suggesting a regional trend toward adjudication-friendly practices ( AlSuwaidi, 2022). Nevertheless, the enduring nationality of agents suggests that the UAE is pursuing a veiled liberalization approach rather than the expansive liberalization in Saudi Arabia.

The UK regime, which follows the Commercial Agents (Council Directive) Regulations 1993, adopts a different philosophy based on the principles of EU Directive 86/653/EEC. UK legislation gives greater importance to the freedom of contract, with fewer registration requirements and lax termination conditions, but also provides indemnity or compensation under Regulation 17 to terminated agents. Case law such as Lonsdale v. Howard & Hallam Ltd [2007] UKHL 32 delivers information on how balancing agent protection and the liberalism of the market, which guided the court in this case, can provide insight to the Saudi policymakers, who want to find a balance between statutory regulation and free market ( Bird & Bird, 2020).

Hybridization can be observed in reforms in Saudi Arabia: protection for agents similar to those in the UAE is applied alongside UK-style arbitration acceptance and termination flexibility. This selective adoption implies purposeful conformity with international standards without violating Shariah principles. The incorporation of features from both systems can make Saudi Arabia a legally advanced jurisdiction that attracts foreign investment while preserving its cultural and legal identity. Future research may examine harmonization across the GCC, developing these bilateral comparisons into a regionally integrated, yet globally competitive, commercial law ( OECD, 2023). Moreover, the analysis of case studies in both the UAE and the UK helps understand how reforms in the field of law are changing across various institutional settings. The gradual changes in the UAE underscore the importance of gradual liberalization as a way to reduce the intensity of stakeholder opposition, whereas the adoption of judicial influence in the UK points to courts as the formers of commercial agency norms ( KPMG, 2023).

A different applicable area is the divergent dispute settlement models. The hybrid model offered by the integrated arbitration provisions of the UAE and ministerial supervision is compared with the UK, which offers the separation of judicial enforcement of the agency’s rights. This comparison provides insight into Saudi Arabia’s efforts to establish a balance between administrative regulation and judicial independence ( Bird & Bird, 2020). Further, the experience of the UK, where the agency principles derived under the EU were retained post-Brexit, indicates the endurance of legal regimes despite political change, which is encouraging to investors and sends a message of resilience in the face of external shocks ( Klein, 2022). This stability would guide Saudi Arabia’s future reforms in the event of a policy change. Lastly, the combination of these comparative lessons helps to better understand the process of hybridization in Saudi Arabia and how the selective incorporation of the features of the two systems, like adjudication mechanisms in the UAE and contract freedoms in the UK, can be used to create a reform model that is locally-oriented and competitive on the international level.

Why these features? A political-economic interpretation of selective borrowing. Saudi decisions are guided by a pragmatic calculus of the 2030 Vision. Arbitration recognition and digital registration are inexpensive, high signal reforms, which decrease frictions in transactions and are a credible commitment to enforce contracts, as is expected by foreign investors. In comparison, domestically accommodating stakeholders by retaining calibrated agent protections, especially compensation in situations of unjustified termination, is in line with domestic stakeholder accommodation in incumbent distributors and SMEs that prioritize relational stability. The outcome is hybridization, where KSA takes the predictability apparatus (registries, more explicit termination rules, ADR) of liberal systems but maintains stability devices known to regional practice. This choice also helps minimize the risk of transplant failure by aligning imported mechanisms with adjudicatory capacity and Shariah-based legitimacy. Judicial art and regulatory advice will play a significant role in converting the borrowed elements into a permanent practice.

### 4.1 Comparative framework: Contextual adaptation and legal alignment

Contextual adjustment rather than wholesale transplants characterizes the commercial agency law reform process in Saudi Arabia. The modernization of the framework has been carried out by Saudi policymakers, using mechanisms that enhance efficiency, predictability, and transparency without affecting the Kingdom’s overall consistency with its legal traditions and Shariah-based principles. These reforms were not borrowed but developed internally and contextualized in line with regional and international experience to be compatible with national priorities and institutional realities. This sentiment echoes the comparative law theory of functional adaptation, which focuses on reforms with a higher success rate when they are tuned to local legal culture, institutional capacity, and normative values. The example of Saudi Arabia shows that successful modernization can be developed within the country’s boundaries, relying on comparative knowledge only as a reference, not as a pattern. This has been guided by the principle of enhancing legal clarity and procedural coherence within a framework grounded in Saudi Arabia’s jurisprudential identity.

In comparison, the model of the Kingdom emphasizes that it is possible to balance domestic goals with international standards in a way that does not violate autonomy but guarantees long-term reform. To illustrate, as other jurisdictions can stress the liberalization of contracts or administrative independence, reforms in Saudi Arabia are oriented towards being structurally predictable, digitally governed, and expanding dispute-resolution strategies in line with national judicial development. The balance achieved explains how the reform may advance modernization objectives without undermining the integrity of the domestic legal system. The analytical construct used in this comparison is therefore based on three principal vectors: 1. Institutional Fit: it is necessary to make sure that regulatory innovations fit in the administrative and judicial capabilities of the Kingdom. 2. Doctrinal Compatibility: complete consistency with Shariah and national law doctrine, and in improving the clarity and predictability of enforcement. 3. Sustainability and Vision 2030 Alignment: entrenching the reforms in the long-term strategic goals of the economic diversification, legal transparency, and good governance.

Overall, the reforms of the Commercial Agency Law in Saudi Arabia are an example of a distinctively moderate modernization process that selectively relies on comparative experience to benchmark, but which is rooted squarely in national legal reasoning, Shariah legitimacy, and institutional continuity. Let us see now this table: Old vs. New Regime Comparison (Illustrative Case).

**Table:** 

Topic	Pre-Reform Provision (1962 Law)	Reformed Provision (2022–2023 Law)	Doctrinal/Practical Effect
Registration	Limited to Saudi nationals only; manual filing required	Allows certain mixed-ownership entities; fully digital registration	Broadens participation; reduces administrative delay
Termination	Vague criteria; heavy bias toward agent compensation	Explicit grounds: expiry, breach, mutual consent; defined compensation	Aligns with fasakh and gharar doctrines; enhances predictability
Dispute Resolution	Courts only; arbitration not recognized	Arbitration expressly permitted (Art. 14); SCCA included	Introduces enforceable ADR aligned with Vision 2030
Transparency	No public registry	Online registry with periodic compliance reporting	Promotes oversight and market trust

## 5. Impact on Foreign Direct Investment (FDI)

It is expected that the reform of the Saudi Arabia Commercial Agency Law will substantially transform the investment environment by minimizing past obstacles and serve as an indicator of regulatory reform. First, greater investor confidence can be achieved through stronger statutory provisions, such as transparent registration (Article 7) and termination (Article 10) rules, which eliminate the perceived legal uncertainty that is one of the main deterrents cited in investment reports ( Sornarajah, 2021).

Second, now, foreign principals have more strategic choices to enter the market through the introduction of flexible agency arrangements, such as non-exclusive and sector-specific arrangements ( O’Kane, 2013). The initial signs of the reforms already affecting investment decisions are the entry of global pharmaceutical and automotive companies into the market ( Ramady, 2010).

Third, arbitration (Article 14) has enhanced predictability in dispute settlement, encouraging cross-border investment by providing greater certainty about the enforceability of contracts ( Al-Ramahi, 2008; Saleh, 1984). Also, alignment with Saudi Arabia’s bilateral investment treaties (BITs) and WTO obligations strengthens legal compliance with international trade standards, thereby lowering perceived sovereign risk ( Sornarajah, 2021). These alignments make Saudi Arabia competitive as a GCC state and emerging market in the quest to attract high-value FDI ( Ballantyne, 1986).

It should be stressed that the numerical variables provide the picture of correlation, not causation. Although the initial signs indicate an increase in investor sentiment, these changes can only be interpreted as part of a larger set of Vision 2030 reforms, such as the liberalization of macroeconomic policies, the simplification of regulations, and the encouragement of industry-specific investments ( Ramady, 2010). In this connection, the agency law reforms are situated within a broader reform context rather than as a unique or determining factor in FDI performance.

Finally, such reforms indirectly strengthen competition at home. With a decline in agency exclusivity, principals could encourage performance-based representation, thereby increasing efficiency in the distribution network ( Alotaibi, 2022). Although encouraging, the long-term effect of FDI will remain unstable unless judicial enforcement, transparency in administration, and perceptions towards investors are maintained ( Foster, 2010). This highlights the importance of longitudinal information and further empirical assessment of the reform efficacy in the dynamic legal-economic environment in Saudi Arabia ( Cane & Kritzer, 2010).

The psychological aspect of investor confidence cannot be underrated. When foreign investors are making decisions, they usually consider not the formal legal framework but the perception of legal predictability and enforceability ( Sornarajah, 2021). Comprehensive legal reform, especially in the areas of digitalization and arbitration recognition, and in accordance with international standards, has symbolic value that can be more significant than the substantive provisions themselves ( Khan, 2010). Saudi Arabia can improve its image as a serious, reliable, and forward-looking investment destination by demonstrating its commitment to modernizing the law ( Ramady, 2010).

Besides, the reforms promote more advanced market-entry strategies. In the past, foreign principals could do little more than appoint a single exclusive agent who served the entire Saudi market, which was frequently inefficient due to the Kingdom’s extensive geographical coverage and diverse regional markets ( O’Kane, 2013). The opportunity to have multiple non-exclusive agents or agents in specific sectors would provide a broader range of distribution policies, a more comprehensive market, and more competition among agents, all of which are beneficial to both the principal and the consumer ( Beraudo, 2014).

Arbitration clauses are vital for FDI. International investors usually prefer arbitration over local court litigation for the following reasons: it is neutral, confidential, expert, and enforceable under the New York Convention ( Saleh, 1984; Yasin, 2012). The explicit acknowledgment and creation of avenues for arbitration enable Saudi Arabia to eliminate one of the most significant impediments to foreign investment and put the country on par with international best practices in commercial dispute resolution ( Al-Ramahi, 2008).

Moreover, aligning the reforms with WTO requirements and bilateral investment treaties minimizes political risk. International law provides investors with the expectation of additional protection through domestic remedies, enabling the establishment of several layers of legal protection ( Sornarajah, 2021). This host protection system is especially appealing to risk-averse institutional investors, including pension funds and sovereign wealth funds, who are demanding high levels of legal certainty before investing institutionally in emerging markets ( Khan, 2010).

The competition that the reforms brought about is also worth considering. The reforms introduce market pressures that encourage agents to improve the quality and reduce the cost of their services and to develop innovative distribution methods ( Alotaibi, 2022). This competition advantage not only benefitsbenefits foreign principals but also Saudi consumers, who can enjoy a broader range of products at more competitive prices. The resulting efficiency will contribute to higher consumer welfare, market dynamism, and allocative efficiency ( Ramady, 2010).

## 6. Challenges and future directions

Despite the considerable changes proposed by the 2022-2023 reforms, several issues may affect their future effectiveness. To begin with, it requires proper implementation, with the judicial application consistent throughout. Inconsistency in judicial experience and understanding of commercial law can lead to different interpretations of the new provisions, causing uncertainty for investors ( Foster, 2010). These risks may be reduced by providing targeted judicial education and by establishing commercial courts that ensure uniformity in judgments ( Cane & Kritzer, 2010).

Second, another challenge is the ingrained opposition of the institutional commercial agents. Reforms might be opposed by many long-term agents who will sue or lobby politically ( O’Kane, 2013). Law transition studies have shown that this boxing may slow the results of reform unless it is accompanied by a well-developed stakeholder engagement and transitional support systems ( Sfeir, 2007).

Third, the more thorough incorporation of arbitration into the framework of commercial dispute resolution provokes the cultural and doctrinal issues. Although this increased caseload is encouraging, doubts remain about the enforcement of foreign arbitral awards, especially those inconsistent with public policy or Shariah ( Saleh, 1984). It would help establish stronger judicial precedents on such matters and make Saudi Arabia a more favorable destination for arbitration ( Al-Ramahi, 2008; Yasin, 2012).

Also, business guidance is not fully available. The lack of standardized model contracts and authoritative commentary regarding the new law puts parties in the position of making interpretive mistakes and drafting flaws ( Beraudo, 2014). Guidelines and explanatory notes from the Ministry of Commerce should also be published to encourage compliance and minimize the number of disputes arising from contract uncertainties ( Foster, 2010).

Lastly, regional integration by the GCC is both a challenge and an opportunity. Cross-border investment strategies are complicated by divergent commercial agency laws among GCC members ( Ballantyne, 1986). Jurisdictional amphetamines would create a common market in the region, enhancing global competitiveness for Gulf trade ( Ramady, 2010). The leadership role of Saudi Arabia in this respect would make it a potential instigator of overall legal integration in the region ( Otto, 2010).

Overall, these issues will be tackled through long-term institutional investment, building judicial capacity, and stakeholder dialogue, along with continuous policy improvement to ensure that the reforms have a transformative effect. In addition, the need to increase legal practitioners’ training, especially those operating in non-commercial hubs, is pressing to ensure that law reform is applied uniformly in the Kingdom ( Cane & Kritzer, 2010). The absence of such initiatives means that interpretation differences might lead to a lack of investor confidence and regional differences in enforcement ( Foster, 2010).

Further, the government should focus on campaigns to raise awareness among the population to advise businesses (particularly SMEs) of their rights and responsibilities under the new law ( O’Kane, 2013). Easy-to-understand materials can also bridge knowledge gaps and address inadvertent non-compliance, making the reforms overall more effective ( Ramady, 2010).

### 6.1 Judicial reconciliation with Shariah doctrines

Saudi adjudication has long mediated commercial change by Shariah-based methods. Three of them are particularly relevant to agency-related disputes: (1) gharar (excessive uncertainty) – courts police indefinite obligations and open-ended penalties; (2) fasakh (rescission) – equitable unwinding in case of defects that hinder the lawful purpose of the contract; and (3) akl al-maal bil-batail (unjust enrichment) – limits windfall compensation in case of defects that are not the purpose of the contract ( Hallaq, 2009; Nyazee, 1998).

Under the reformed law, we anticipate no conflict but rather harmonizing moves: we permit judges to apply purposive reasoning (maqsimad al-sharik) and proportionality to relate termination and compensation clauses, thereby avoiding oppressive results without compromising the predictability of the statute ( El-Gamal, 2006). The prospect of such harmonization leads to drafting (clarity of scopes/terms), disclosures of registration, and liquidated damages based on verifiable metrics, which reduce the risk of litigation in a Shariah-consistent way ( Vogel & Hayes, 1998).

The three doctrines map to recurring contractual problems in agency arrangements. Specifically, the doctrine of gharar applies to contracts of indefinite nature, where there is no end, open obligations, and performance limits defined in a vague manner ( El-Gamal, 2006). The reason is that courts have traditionally restricted compensation claims when the agency terms underlying the claim posed too much uncertainty. Fasakh doctrine is most frequently used in cases of material defects, performance failure, or failure of a contract to fulfill its purpose in accordance with the law, providing an opportunity to unwind the agreement on equitable terms that do not imply punitive action ( Nyazee, 1998). Akl al-m2al bil-b2til (unjust enrichment) is, in the meantime, implicated in agency contracts that include punitive liquidated damages, unreasonable compensation, or exclusivity deals that provide windfall benefits without any reasonable business explanation ( Hallaq, 2009).

The debate of judicial reasoning is based on two levels of analysis. To begin with, there is already documented experience of Saudi judges using proportionality and uncertainty-driven reasoning to trim excessive claims, even in the context of contractual ambiguity combined with Shariah maxims ( Alzahrani, 2024). Second, our anticipations about the use of the reformed statutory provisions are prospective and based on perceived patterns of adjudication, rather than claimed empirical certainties ( Foster, 2010). Since the post-2022 regime is still in its early stages, these forecasts should be treated as analytical possibilities derived from the existing doctrinal toolkit of the Saudi courts rather than forecasts ( Hallaq, 2009).

The other significant problem is integrating technology-backed compliance tools. Digitization has simplified the registration process, but more sophisticated systems for monitoring and tracking disputes during contract execution might enhance transparency and reduce administrative work ( Ramady, 2010). Arbitral awards should also be enforced across borders. Saudi Arabia has signed the New York Convention, but specific opposition to enforcement based on Shariah remains ( Saleh, 1984). To ensure that Saudi Arabia remains viewed as an arbitration-friendly jurisdiction, establishing stable rules for such reviews will be essential ( Al-Ramahi, 2008; Yasin, 2012).

In addition, engaging with foreign chambers of commerce and investor councils may provide policymakers with effective feedback on the challenges of reform implementation and good practices, thereby establishing a responsive policy environment ( Sornarajah, 2021). The reforms also require greater cooperation with academic institutions to produce empirical studies of judicial trends, their effects on investments, and industry-specific effects ( Cane & Kritzer, 2010). There will be a need for data-driven policy making to achieve refinements and long-term success ( Foster, 2010).

Lastly, incorporating such legal changes into broader regional economic projects, such as GCC market harmonization and free trade agreements, would not only strengthen the Saudi Arabian legal system but also increase the overall competitiveness of the GCC region in the eyes of international capital ( Ballantyne, 1986; Ramady, 2010). These interpretation channels, combined with the principles of fiqh al-muqamanalaat, which focus on clarity, fairness, and balance in commercial transactions, and the larger maqashid al-sharigah, that is, the protection of wealth, the avoidance of harm, and the upholding of contractual justice ( Vogel & Hayes, 1998). By pegging judicial expectations to these solid juristic structures, it is possible to ensure that statutory reforms develop coherently within the normative tradition of Saudi Arabia and provide predictability to contemporary commercial practice ( Hallaq, 2009; Nyazee, 1998).

## 7. Conclusion

The 2022-2023 changes to the Saudi Arabia Commercial Agency Law were a historic legislative change in the Kingdom that will make domestic regulations and global trade standards consistent, while the core shall remain Shariah-compliant. These reforms break the century-old protectionist barriers, improve investor confidence through more transparent rules and electronicized processes, and boost dispute-resolution systems by formally recognizing arbitration ( Al-Ramahi, 2008; Saleh, 1984). Each of them is an indicator of a more drastic move towards an open, predictable, and investment-friendly business climate in line with Vision 2030 ( Ramady, 2010).

As noted earlier in this paper, the pre-reform regime was concerned with protecting local agents, yet it unintentionally restricted market entry and hindered FDI ( O’Kane, 2013). Saudi Arabia has shown its interest in legal modernization and economic diversification by acknowledging reforms that align the rights of agents with contract autonomy and best practices worldwide ( Sornarajah, 2021). Although the reforms reinforce the doctrinal framework of agency law and align the statutory mechanisms with international best practices, the overall economic implications should be considered with a grain of salt. The first signs of a better investor attitude are indicative but not definitive since several Vision 2030 projects are running in tandem ( Ramady, 2010). In this regard, the role of the reform in investor confidence should be viewed as a supportive factor among the many parallel legal and economic trends.

The comparative analysis of the UAE and the UK points to a hybrid nature in Saudi Arabia, where it selectively adopts liberalizing attributes while retaining its legal identity grounded in Islamic principles ( Hallaq, 2009; Otto, 2010). However, practical judicial interpretation, inter-regional consistency, strong regulation, and ongoing consultations with the stakeholders will ensure the successful implementation of these reforms ( Foster, 2010). The issue of implementation, including judicial training to counter entrenched agents, will play a central role in solidifying the reform’s returns and creating a transparent and competitive commercial market ( Cane & Kritzer, 2010).

The empirical conclusions in the paper are speculative. The changing investment environment in Saudi Arabia is due to several simultaneous reforms, and amendments to the agency law are only one strand of the broader changes underway ( Ramady, 2010). The trends observed must thus be viewed as early warning signs other than hard evidence of causal effect.

To sum up, the commercial agency reform system in Saudi Arabia not only reinforces the local legal framework but also enhances its appeal as a commercial and investment destination within the region. The assertions about Saudi Arabia’s development as a regional business center must thus be viewed as informed speculation based on the legal path rather than conclusive empirical evidence. These expectations may be justified by the observable correlation between the reforms and the chosen investment indicators, but additional time-series data are needed to determine causality ( Cane & Kritzer, 2010). Future studies on investor behavior, the consequences of contract enforcement, and industry-specific patterns of dispute resolution will play a crucial role in confirming the long-term impacts of the reforms on FDI ( Sornarajah, 2021).

The following study should be an empirical investigation of the economic consequences of the reforms, long-held investor attitudes, and prospects for legal harmonization across the GCC ( Ballantyne, 1986). The Kingdom can serve as a valuable example of how to combine contemporary commercial law with Shariah within the global legal system by maintaining momentum in legal reform and keeping pace with international standards ( Hallaq, 2009; Vogel & Hayes, 1998).

Besides, such reforms serve as an example for other emerging markets that struggle to harmonize local legal traditions with the demands of international trade ( Otto, 2010). Researching the experience in Saudi Arabia, other policymakers can learn how to sequence reform, involve stakeholders, and introduce legal predictability into culturally based legal frameworks ( Reimann & Zimmermann, 2019).

The findings of this analysis also emphasize the need for repeated evaluation. Empirical research on judicial rulings, the rate of contract enforcement, and indicators of investor reaction will play a critical role in determining the effectiveness of reforms and in applying fine-tuning measures as they mature ( Foster, 2010). Moreover, the courtship between governmental organizations, non-governmental parties, and scholars should be maintained to create an informed, evidence-based future of legal innovations ( Cane & Kritzer, 2010). This will make reform benefits broadly known and applied across all economic sectors.

Finally, embed these reforms within a broader regional integration framework to enhance their effectiveness. It is possible to improve the state’s legal and business competitiveness by supporting the harmonization of agency legislation across the GCC and by cooperating with neighbors to facilitate the ambitions of Vision 2030 programs ( Ramady, 2010).

## 8. Policy recommendation summary

Cluster 1 — Judicial & institutional capacity.

(a) Specialized training for commercial judges and neutrals on termination standards, compensation calibration, and arbitral-award enforcement in a *Shariah* context.

(b) Curated **guidance notes** (benchbooks, circulars) to standardize interpretation and reduce regional variability.

Cluster 2 — Regulatory transparency & compliance tooling.

(a) Public **model clauses/contracts** for common agency forms (exclusive/non-exclusive; sectoral variants), annotated for *Shariah* compatibility.

(b) A dashboard within the Ministry’s portal offering renewal alerts, standardized termination notices, and anonymized dispute statistics for market transparency.

Cluster 3 — Regional harmonization & investor engagement.

(a) GCC-level dialogue on registration interoperability, minimum termination standards, and recognition of ADR outcomes.

(b) Periodic consultation with foreign and domestic investors to iterate guidance based on frictions encountered in contracting and enforcement.

These clusters derive directly from the doctrinal and comparative analysis: predictable adjudication (Cluster 1) gives effect to the statutory design; transparent tooling (Cluster 2) reduces transaction costs; regional convergence (Cluster 3) scales investor certainty across the Gulf.

## Data Availability

No data is associated with this article. No new proprietary dataset was generated for this study. All numerical indicators and empirical references are drawn from publicly available official reports issued by the General Authority for Statistics, the Ministry of Commerce, the Saudi Center for Commercial Arbitration (SCCA), and the World Bank. A full list of quantitative indicators, including exact years and sources, is provided in Appendix A1. Extended data, including Appendix A1 containing all numerical indicators and their sources, are openly available on Figshare at: https://doi.org/10.6084/m9.figshare.30740285.v1 ( Alotaibi, 2025). Data are available under the terms of the Creative Commons Attribution 4.0 International License (CC BY 4.0).
