# Supplementary material for: Modernizing Commercial Agency Regulations in Saudi Arabia: Legal Reforms and Comparative Insights
Source: F1000Res. 2026 Jan 21;14:912. Originally published 2025 Sep 12. [Version 4] doi: 10.12688/f1000research.168970.4 (PMC12673251; doi:10.12688/f1000research.168970.4)
Supplement: Peer review history

# Reviewer report: Reviewer response for version 3

**DOI:** 10.5256/f1000research.192338.r438279

**Published:** 2025-12-28

**Authors:** Mohamad Hidayat Muhtar

**Affiliations:** 1 Universitas Negeri Gorontalo, Gorontalo, Indonesia

I have carefully reviewed the revised manuscript and the authors’ detailed point-by-point response. In my view, the revisions directly and substantively address the concerns raised in my initial report. The exposition has been tightened, with reduced repetition around the themes of digitisation, arbitration, and the shift toward party autonomy, and the argument now progresses more clearly across the sections. The literature base has been strengthened through the incorporation of additional academic work on agency law, distributorship, legal transplants, and Middle Eastern commercial law, so that practitioner and policy materials are now used primarily for contextualisation rather than for conceptual support. The methodological underpinnings of the comparative design have been clarified: the rationale for selecting the UAE and the UK as comparator jurisdictions is now explicit, and the axes along which the comparison is operationalised are clearly set out.

 The revised Section 1.1 now functions more transparently as a methods subsection. It specifies the principal statutes and regulations examined, explains how administrative circulars were identified, and clarifies that the case-law discussion is illustrative rather than exhaustive. This significantly improves methodological transparency and replicability. The treatment of quantitative indicators has also been refined. The authors have moderated the causal language associated with FDI, registration, and business-environment metrics, situating any observed improvements within the broader context of concurrent Vision 2030 reforms and macroeconomic developments. An appendix table documenting each indicator, with year, value, and precise source, together with a corrected data-availability statement, brings the empirical component to an acceptable standard of transparency.

 Overall, the conclusions regarding doctrinal and institutional change remain well grounded in the statutory and comparative analysis, while broader claims about economic impact and investor behaviour have been appropriately reframed as cautious expectations or hypotheses for future empirical testing. 

 Taken together, these revisions materially enhance the clarity, methodological robustness, and balance of the article. I am satisfied that the manuscript now meets the scientific and scholarly standards required for indexing and I have no further substantive comments.

Is the work clearly and accurately presented and does it cite the current literature?

Partly

If applicable, is the statistical analysis and its interpretation appropriate?

Not applicable

Are all the source data underlying the results available to ensure full reproducibility?

Partly

Is the study design appropriate and is the work technically sound?

Yes

Are the conclusions drawn adequately supported by the results?

Partly

Are sufficient details of methods and analysis provided to allow replication by others?

Partly

Reviewer Expertise:

Legal Reform,Legal Research Methodology; Evidence-Based Policy Development, comparative law, commercial law

I confirm that I have read this submission and believe that I have an appropriate level of expertise to confirm that it is of an acceptable scientific standard.

# Reviewer report: Reviewer response for version 3

**DOI:** 10.5256/f1000research.192338.r438278

**Published:** 2025-12-10

**Authors:** Sabri Boubaker

**Affiliations:** 1 EM Normandie Business School, Métis Lab, Caen, France

I am happy with the current version of the paper.

Is the work clearly and accurately presented and does it cite the current literature?

Yes

If applicable, is the statistical analysis and its interpretation appropriate?

Not applicable

Are all the source data underlying the results available to ensure full reproducibility?

Yes

Is the study design appropriate and is the work technically sound?

Yes

Are the conclusions drawn adequately supported by the results?

Yes

Are sufficient details of methods and analysis provided to allow replication by others?

Partly

Reviewer Expertise:

Managment and finance

I confirm that I have read this submission and believe that I have an appropriate level of expertise to confirm that it is of an acceptable scientific standard.

# Reviewer report: Reviewer response for version 2

**DOI:** 10.5256/f1000research.191347.r433824

**Published:** 2025-12-02

**Authors:** Farhaan Wali

**Affiliations:** 1 Bangor University, Bangor, UK

I am happy with the amendments undertaken, as they enhance the scope and methodological foundation of the article.

Is the work clearly and accurately presented and does it cite the current literature?

No

If applicable, is the statistical analysis and its interpretation appropriate?

Yes

Are all the source data underlying the results available to ensure full reproducibility?

Yes

Is the study design appropriate and is the work technically sound?

Yes

Are the conclusions drawn adequately supported by the results?

Yes

Are sufficient details of methods and analysis provided to allow replication by others?

Yes

Reviewer Expertise:

Islamic Studies, Islam in Britain, Islamism and radicalisation.

I confirm that I have read this submission and believe that I have an appropriate level of expertise to confirm that it is of an acceptable scientific standard.

# Reviewer report: Reviewer response for version 2

**DOI:** 10.5256/f1000research.191347.r433825

**Published:** 2025-12-02

**Authors:** Alija Avdukic

**Affiliations:** 1 University of Dundee, Dundee, UK

Many thanks for revising you manuscript. I have no further comments. Best wishes.

Is the work clearly and accurately presented and does it cite the current literature?

Partly

If applicable, is the statistical analysis and its interpretation appropriate?

Not applicable

Are all the source data underlying the results available to ensure full reproducibility?

Partly

Is the study design appropriate and is the work technically sound?

Partly

Are the conclusions drawn adequately supported by the results?

Partly

Are sufficient details of methods and analysis provided to allow replication by others?

No

Reviewer Expertise:

Political economy and Islamic Finance.

I confirm that I have read this submission and believe that I have an appropriate level of expertise to confirm that it is of an acceptable scientific standard.

# Reviewer report: Reviewer response for version 2

**DOI:** 10.5256/f1000research.191347.r434666

**Published:** 2025-11-25

**Authors:** Mohamad Hidayat Muhtar

**Affiliations:** 1 Universitas Negeri Gorontalo, Gorontalo, Indonesia

This article charts the recent reform of Saudi Arabian agency and distribution law and places those changes against a wider comparative and law-and-development background. The authors explain how the new statutory regime restructures registration, relaxes nationality requirements, clarifies termination and compensation, and incorporates greater scope for arbitration. The narrative links these developments to Vision 2030 and to ambitions for Saudi Arabia to function as a regional hub for commercial intermediation. A conceptual framing based on legal transplants, institutional adaptation, and Sharia-based principles such as gharar, fasakh and protection against unjust enrichment underpins the argument. Comparative references to UAE and UK law are used to illustrate possible models and to highlight the distinctiveness of the Saudi pathway.

 The exposition is generally clear and the paper is well organised into background, pre-reform framework, description of the new regime, comparative analysis and policy implications. The conceptual section and the Sharia discussion give the work an intellectual backbone that distinguishes it from purely descriptive practitioner pieces. Some tightening would still help. Themes such as digitisation of registration, expansion of arbitration, and the shift from rigid mandatory rules to more flexible party autonomy appear in several sections with similar wording and limited new insight. Streamlining those passages and using sharper signposting of what each section adds would make the central line of argument easier to follow and would remove a lingering sense of repetition. The literature base is adequate and reasonably up-to-date, although reliance on policy reports and law-firm memoranda is relatively high. Incorporating more academic work on agency and distributorship, legal transplants, and Middle Eastern commercial law would situate the contribution more firmly within existing scholarship and not only within policy debate.

 The study design is appropriate for the questions posed. A doctrinal and comparative approach suits an inquiry into how statutory reform restructures commercial relationships and how that reform interacts with Sharia and investment policy. The choice of UAE and UK law as comparators is defensible: one offers a nearby civil-law and regional benchmark, the other a liberal common-law reference point attractive to foreign investors. The way in which the article draws out convergence and divergence across these systems is generally persuasive, and the hybrid character of the Saudi reform is convincingly described. That said, the reasoning behind comparator selection remains somewhat implicit. A compact methodological explanation of why these two jurisdictions were chosen over others in the region or beyond, and how the comparison was operationalised, would anchor the design more firmly.

 Methodological transparency and replicability could be improved. The article states the period covered and lists broad categories of sources, including legislation, implementing regulations, administrative circulars, case law, policy documents and investment data. Readers still have to infer how specific materials entered the analysis. A short dedicated methods subsection would be valuable. That section could identify the principal statutes and regulations examined, spell out how administrative instruments and court decisions were selected, and clarify whether the case law discussion is illustrative or based on a more systematic search. A more explicit account would allow future researchers to replicate or extend the study and would enhance confidence in the coverage of sources.

 Quantitative evidence is used sparingly yet plays a visible role in linking legal reform to policy outcomes. References to growth in FDI, numbers of registered agency agreements and selected business-environment indicators give useful context, and the article does not attempt sophisticated statistical modelling, which is not necessary for this type of work. Interpretation of these figures sometimes edges toward stronger causal claims than the underlying data can support. Several passages appear to move from temporal coincidence between reforms and improved indicators toward a narrative in which the new agency law is portrayed as a key driver of investment attraction. A more cautious tone would increase robustness. Phrasing that stresses correlation, acknowledges the presence of concurrent reforms and macroeconomic changes, and frames the legal changes as one contributing factor rather than the decisive cause would align the conclusions with the evidentiary base.

 Data availability is partly satisfactory. The primary legal materials on which the doctrinal analysis rests are public and citable. Many of the quantitative claims draw on official or well-known international sources. The statement that no data are associated with the article feels slightly at odds with the use of specific numerical indicators. Readers interested in verifying or reusing those figures would benefit from a short table or appendix that lists each indicator, its value, year and precise source. Such documentation would be straightforward to produce and would significantly enhance transparency and reproducibility without turning the paper into an empirical economics study.

 The conclusions concerning doctrinal change are, on the whole, well supported. The article demonstrates convincingly that the new regime modernises registration, reduces formal barriers for foreign principals, reorganises termination and compensation, and enlarges scope for arbitration while still embedding protective mechanisms that can be defended in terms of Sharia and public policy. The portrayal of the reform as a hybrid model that blends international investor-friendly elements with domestic normative commitments follows naturally from the analysis of the statutory text and comparative material. Claims that link these legal changes to wider economic outcomes are more ambitious than the evidence currently allows. Assertions about a decisive boost to investor confidence or a firmly established role for Saudi Arabia as a regional hub are better treated as informed expectations or hypotheses, pending richer empirical backing. Either a more cautious formulation or the inclusion of more granular investment or dispute-resolution data would bring those passages into better proportion with the available evidence.

 Several revisions appear necessary for the article to be scientifically sound in the sense expected of a scholarly legal and policy journal. First, exposition should be tightened by pruning repeated discussions of the same theme and clarifying what each section uniquely contributes. Second, methodology and source selection need to be described in a concise yet explicit subsection that identifies the core primary materials, explains the basis for including particular administrative and judicial sources, and justifies the choice of comparator jurisdictions. Third, all quantitative indicators used in the argument should be documented in a table or appendix, and the main text should adopt language that clearly distinguishes correlation from causation. Fourth, conclusions that speak to economic impact and investor behaviour should either be reframed as tentative and exploratory or supported by additional empirical material. Addressing these points would preserve the strengths of the current manuscript,a timely topic, a thoughtful theoretical framing, and a careful engagement with Sharia,while providing a clearer view of how the analysis was constructed and how far the findings can reasonably be taken.

Is the work clearly and accurately presented and does it cite the current literature?

Partly

If applicable, is the statistical analysis and its interpretation appropriate?

Not applicable

Are all the source data underlying the results available to ensure full reproducibility?

Partly

Is the study design appropriate and is the work technically sound?

Yes

Are the conclusions drawn adequately supported by the results?

Partly

Are sufficient details of methods and analysis provided to allow replication by others?

Partly

Reviewer Expertise:

Legal Reform,Legal Research Methodology; Evidence-Based Policy Development, comparative law, commercial law

I confirm that I have read this submission and believe that I have an appropriate level of expertise to confirm that it is of an acceptable scientific standard, however I have significant reservations, as outlined above.

# Response

**Published:** 2025-11-26

**Authors:** Hajed A. Alotaibi

**Affiliations:** Majmaah University, Saudi Arabia

We are grateful for your careful, generous, and insightful report. The comments have helped us refine the structure, methodology, and empirical framing of the article. Below, we respond point-by-point and indicate the corresponding revisions.

**1. Repetition and signposting of themes**

**Comment:** Themes such as digitisation, arbitration, and the shift toward party autonomy are repeated in several sections; sharper signposting would help.

**Response:** We agree and have streamlined the exposition as follows:

- Concentrated the fuller discussion of digitised registration in Section 3 (The 2022–2023 reforms), with only brief reminders in Section 5.
- Centralised the main discussion of arbitration in Sections 3 and 5, and removed repetitive phrasing from Sections 4 and 7.

**2. Literature base: more academic work**

**Comment:** Policy reports and law-firm memoranda are heavily used; more academic literature on agency/distributorship, legal transplants, and Middle Eastern commercial law would help.

**Response:** We appreciate this suggestion and have already:

- Added several references to academic works on agency law, distributorship, and Middle Eastern commercial law (in addition to those already cited).
- Clarified the distinction between policy/practitioner sources (used primarily for context and recent practice) and scholarly sources (used for conceptual framing and doctrinal critique).

**3. Comparator selection (UAE and UK)**

**Comment:** The choice of UAE and UK is defensible but the rationale remains somewhat implicit; a compact explanation of comparator selection and operationalisation is needed.

**Response:** We agree and have made this explicit by:

- Adding a brief methodological justification near the start of Section 4, explaining that the UAE is chosen as a GCC civil-law and regional benchmark, and the UK as a liberal common-law system with an EU-derived agency regime that is attractive to foreign investors.
- Stating that the comparison is operationalised along three dimensions: registration/market access, termination/compensation, and dispute resolution design.

**4. Methodology and source selection**

**Comment:** A dedicated, explicit methods subsection would enhance transparency and replicability.

**Response:** Thank you for this helpful suggestion. We already had Section 1.1 “Methodology and sources”, and we have now strengthened it to function clearly as a methods section by:

- Listing the principal statutes and regulations analyzed (1962 law, 2022–2023 law, implementing regulations, related legislation).
- Clarifying how administrative circulars were included (publicly accessible circulars and directives via the Ministry portal).
- Explaining that the case-law discussion is illustrative rather than exhaustive, based on leading and representative decisions, and that doctrinal trends are supplemented by secondary scholarship.

**5. Quantitative indicators and causality**

**Comment:** FDI, registration, and business-environment indicators are useful, but interpretation sometimes edges toward stronger causal claims than the data support.

**Response:** We fully agree and have:

- Re-phrased the main FDI and business-indicator passages in Section 5 and the Conclusion to emphasize correlation, “early signals,” and “one contributing factor among concurrent reforms” rather than causation.
- Added explicit acknowledgement that multiple Vision 2030 initiatives and macroeconomic policies are unfolding simultaneously, making it difficult to isolate the agency law’s specific causal impact.

**6. Data availability and documentation**

**Comment:** The use of specific numerical indicators sits uneasily with the statement “No data is associated with this article”; a short appendix or table would help.

**Response:** We appreciate this and have revised the manuscript as follows:

- Added a short Appendix / Table A1 listing each quantitative indicator used (FDI growth, number of registered agency contracts, SCCA caseload, Doing Business score changes), together with year, value, and precise source (e.g., General Authority for Statistics, SCCA, World Bank, UNCTAD).
- Revised the Data availability statement to clarify that no new dataset was generated, and that all quantitative indicators are drawn from publicly available official or international reports fully documented in the appendix.

**7. Conclusions and economic impact claims**

**Comment:** Conclusions on doctrinal change are well supported; claims about economic impact and investor behaviour should be more cautious or supported by additional data.

**Response:** Thank you for this nuanced observation. We have:

- Retained the stronger formulation for doctrinal and institutional conclusions, where they directly track the statutory text and comparative analysis.
- Reframed broader economic and investor-behaviour claims in Section 7 (Conclusion) as informed expectations or hypotheses that require further empirical testing. We now explicitly call for future empirical research on investment flows, dispute-resolution trends, and investor perceptions to validate these expectations.

We are thankful for your generous and constructive feedback. Your comments have materially improved the manuscript’s clarity, methodological transparency, and balance between doctrinal analysis and empirical claims.

 Kind regards,

 The Authors

# Reviewer report: Reviewer response for version 2

**DOI:** 10.5256/f1000research.191347.r434663

**Published:** 2025-11-25

**Authors:** Sabri Boubaker

**Affiliations:** 1 EM Normandie Business School, Métis Lab, Caen, France

The topic is timely, policy-relevant, and situated within an important moment of legal transformation under Vision 2030. The manuscript shows significant effort and clear familiarity with the evolving Saudi legal landscape. However, to be suitable for publication, the paper requires substantial conceptual, structural, and methodological refinement. 

 The first major issue concerns the articulation of the manuscript’s core contribution. While the paper is rich in detail, it is not sufficiently clear what the central novelty is in relation to the large body of work on legal transplants, hybrid legal systems, Shariah–civil law harmonization, or agency law reforms in the GCC. The authors gesture toward hybridization and institutional adaptation, yet these concepts have been extensively theorized in comparative law literature. The manuscript would benefit from identifying a single, explicit argument that distinguishes it from prior scholarship—whether that lies in a new typology of reforms, a new doctrinal interpretation, or a new model explaining Shariah–statutory interaction.

 The second major issue relates to the breadth of the manuscript, which dilutes analytical depth. The paper undertakes doctrinal mapping, Shariah jurisprudence, economic impact assessment, empirical illustration, comparative benchmarking, institutional analysis, WTO/BIT alignment, and policy prescription. This breadth creates conceptual diffusion, making it difficult for the reader to grasp the main analytical thread. A more focused approach—with a clearly delimited scope—would allow the authors to develop their arguments more rigorously and avoid repetitiveness.

 The third major concern involves the doctrinal analysis. While statutory provisions are referenced, the interpretation often becomes descriptive rather than analytical. A doctrinal study must do more than summarize statutory changes; it must interpret how these provisions function in practice, highlight areas of ambiguity, interrogate the coherence of new rules, and examine their likely judicial application. Assertions regarding past judicial behavior or statutory inconsistency should be supported by multiple cases or authoritative sources rather than isolated references.

 The fourth major issue concerns the comparative analysis. Although the UAE and UK are appropriate comparators, the discussion lacks methodological rigor. The comparative exercise moves between topics without a consistent framework, making it difficult to assess the relevance of similarities and divergences. Comparative law scholarship emphasizes structured comparison—based on functional equivalence, institutional capacity, doctrinal architecture, or regulatory purpose. A more disciplined comparative method would substantially strengthen the value of this section.

 The fifth major issue lies in the treatment of Shariah-based doctrines. The discussion of gharar, fasakh, and unjust enrichment is valuable, but it is not sufficiently theorized or empirically grounded. The paper should clarify the extent to which these doctrines actively shape judicial decision-making in commercial agency disputes in contemporary Saudi practice. Without deeper engagement with fiqh al-muʿāmalāt, maqāṣid al-sharīʿa, and contemporary Saudi jurisprudence, some interpretive claims risk appearing speculative. Demonstrating doctrinal interaction through a broader set of cases would strengthen the argument.

 The sixth major concern relates to empirical claims. The manuscript cites numerical indicators (FDI increases, SCCA caseload growth, registration data) but does not provide methodological transparency or a clear analytical framework for assessing causality or correlation. Early descriptive signals should not be overstated. A stronger empirical section would specify data sources, clarify the limitations of attributing economic changes to legal reform, and avoid normative or promotional tone. Without such methodological grounding, empirical assertions weaken rather than reinforce the analysis.

 In addition to these major concerns, I provide the following minor comments, which focus on clarity, rigor, style, and consistency. Some sections include very long paragraphs—consider improving readability through sub-sections. Ensure consistent citation format throughout. Avoid overly long sentences that obscure key points. Clarify acronyms at first use (BITs, ADR, SCCA, etc.). Remove duplicate information on FDI increases and SCCA statistics. Ensure consistency in transliteration of Arabic terms (e.g., akl al-māl bil-bāṭil). Revisit claims about judicial inconsistency—support with case evidence. The table “Old vs. New Regime” is referenced but not shown—add it or remove the reference.

 In its current form, the manuscript requires minor revision. With a more focused structure, sharper articulation of originality, more rigorous doctrinal and comparative analysis, and more disciplined treatment of empirical evidence, the paper could make a meaningful contribution to the study of commercial law reform in the GCC. I encourage the authors to undertake a major restructuring of the paper to bring its considerable material into a clearer and more compelling analytical form.

Is the work clearly and accurately presented and does it cite the current literature?

Yes

If applicable, is the statistical analysis and its interpretation appropriate?

Not applicable

Are all the source data underlying the results available to ensure full reproducibility?

Yes

Is the study design appropriate and is the work technically sound?

Yes

Are the conclusions drawn adequately supported by the results?

Yes

Are sufficient details of methods and analysis provided to allow replication by others?

Partly

Reviewer Expertise:

Managment and finance

I confirm that I have read this submission and believe that I have an appropriate level of expertise to confirm that it is of an acceptable scientific standard, however I have significant reservations, as outlined above.

# Response

**Published:** 2025-11-26

**Authors:** Hajed A. Alotaibi

**Affiliations:** Majmaah University, Saudi Arabia

We are grateful for the careful reading of our manuscript and for the thoughtful and constructive comments. The report has been extremely helpful in sharpening the focus, structure, and methodological clarity of the article. Below, we respond point-by-point and indicate how we have revised the manuscript accordingly.

**1. Core contribution and originality**

**Comment:** The central novelty relative to existing work on legal transplants, hybrid systems, Shariah–civil law harmonisation, and GCC agency law reforms is not sufficiently clear.

**Response:** Thank you for highlighting the need to sharpen the core contribution. We have now added an explicit statement of originality at the end of the Introduction, immediately before the “Conceptual framing” subsection.

- What we added: 2–3 sentences clarifying that the article’s main contribution is:
  - (i) proposing a three-vector framework (institutional fit, doctrinal compatibility, Vision 2030 alignment) for evaluating commercial agency reforms in a Shariah-grounded system; and
  - (ii) showing how doctrinal recalibration at the intersection of agency law and investment policy produces a distinctive hybrid model that differs from both UAE and UK approaches and from existing transplant literature.

**2. Breadth vs analytical depth**

**Comment:** The manuscript covers many dimensions (doctrinal, Shariah, economic, empirical, comparative, institutional, WTO/BIT, policy), which risks conceptual diffusion and repetition.

**Response:** We agree that focus improves clarity. We have therefore:

- Clarified the primary scope in Section 1.2 Objectives by explicitly stating that the core focus is doctrinal and comparative analysis of the 2022–2023 reforms, with economic and policy aspects treated as supporting context rather than as co-equal research pillars.
- Streamlined overlapping material by:
  - pruning repeated references to digitisation, arbitration and Vision 2030 in Sections 3, 4, 5, and 7, and
  - tightening the WTO/BIT discussion in the Introduction to one concise paragraph less focused on general law-and-development rhetoric.

**3. Doctrinal analysis: from description to interpretation**

**Comment:** Doctrinal analysis is sometimes descriptive; more interpretive work is needed, supported by more than isolated cases.

**Response:** We appreciate this important point and have strengthened doctrinal analysis by:

1. Expanding interpretation of key provisions in Section 3 (The 2022–2023 reforms):
  - After describing each “axis” (market access, digital registration, contract governance, dispute resolution), we now add 1–2 sentences assessing likely areas of ambiguity and how courts may interpret these rules in practice.
2. Clarifying case-law basis in the Pre-reform section (Section 2) and in Section 6.1:
  - We now explicitly state that the Board of Grievances case No. 2451/1435H is used as an illustrative example of compensation narrowing.
  - Where we mention judicial inconsistency, we have softened the wording and linked it to Alayed et al. (2025) and other secondary sources instead of presenting it as a broad empirical generalisation.

**4. Comparative analysis methodology**

**Comment:** The comparative analysis (UAE/UK) needs a more consistent framework.

**Response:** We agree and have made the comparative method more explicit by:

- Adding a short methodological paragraph at the start of Section 4 (Comparative analysis) explaining that the comparison is structured along three dimensions:
  1. regulatory treatment of registration and market access;
  2. termination and compensation rules;
  3. dispute-resolution design (litigation vs arbitration).
- Ensuring that the UAE and UK subsections follow this same sequence so that similarities and divergences can be read functionally.

**5. Treatment of Shariah doctrines**

**Comment:** Discussion of *gharar*, *fasakh*, and unjust enrichment needs deeper, less speculative engagement with fiqh and contemporary practice.

**Response:** We are grateful for this guidance. We have:

- Expanded Section 6.1 “Judicial reconciliation with Shariah doctrines” by:
  - briefly linking each doctrine ( *gharar*, *fasakh*, *akl al-māl bil-bā* *ṭil*) to specific types of agency clauses (e.g. indefinite duration, punitive compensation, exclusivity); and
  - more clearly distinguishing between documented judicial practice and forward-looking expectations under the reformed regime.
- Added a short reference to fiqh al-muʿāmalāt and maqāṣid al-sharīʿa at the end of Section 6.1 to anchor interpretive expectations in established Shariah scholarship rather than in general assertions.

**6. Empirical claims and methodological transparency**

**Comment:** FDI/registration/SCCA figures lack methodological grounding; causality is sometimes overstated.

**Response:** We fully agree that empirical claims should be modest and transparent. We have therefore:

1. Toned down causal language in Section 5 (Impact on FDI) and in the Conclusion (Section 7):
  - We now speak of “correlation,” “early indicative trends,” and “one contributing factor among several reforms” instead of suggesting that agency law reform is the decisive driver.
  - We explicitly note that multiple Vision 2030 reforms and macroeconomic policies operate simultaneously.
2. Documented indicators more clearly:
  - We have created a short Appendix / Table A1 listing each numerical indicator used (FDI growth, number of registrations, SCCA caseload, Doing Business scores), with year and exact source (e.g. General Authority for Statistics 2023, SCCA 2023, World Bank 2020/2022).
3. Revised the Data Availability statement to clarify that:
  - no new proprietary dataset was generated;
  - all figures are drawn from publicly available official reports and are fully listed in the appendix.

**7. Minor issues (style, consistency, table)**

**Comment:** Long paragraphs, long sentences, acronyms, duplicate FDI/SCCA info, transliteration consistency, judicial inconsistency claims, missing “Old vs New” table.

**Response:** We have addressed these as follows:

- Readability: We have split several long paragraphs in the Introduction, Sections 2, 4, and 5 into shorter ones and simplified overly long sentences.
- Acronyms: All acronyms (BITs, ADR, SCCA, etc.) are now spelled out at first use in the Introduction or Section 5.
- Duplication: We removed duplicated FDI and SCCA statistics, so each numerical claim appears only once in Section 5.
- Transliteration: We standardised *akl al-māl bil-bā* *ṭil* spelling throughout.
- Judicial inconsistency: We softened the language and referenced Alayed et al. (2025) as the main authority rather than making broad empirical claims.
- Old vs New table: The “Old vs New Regime” table is now fully included in Section 4.1 and labelled appropriately.

We hope these revisions address your concerns and enhance the clarity and rigor of the article. We are sincerely thankful for the constructive feedback and believe the manuscript is significantly improved as a result.

 Kind regards,

 The Authors

# Reviewer report: Reviewer response for version 1

**DOI:** 10.5256/f1000research.186220.r414340

**Published:** 2025-10-15

**Authors:** Alija Avdukic

**Affiliations:** 1 University of Dundee, Dundee, UK

The manuscript aspires to provide a doctrinal and comparative analysis of Saudi Arabia’s 2022–2023 commercial agency reforms within the Vision 2030 agenda. In its present form, it reads less like a rigorous academic article and more like an extended policy brief padded with generalities. The argumentation is largely descriptive, repetitious, and insufficiently substantiated. The theoretical apparatus is missing, the comparative component is superficial, the engagement with Shariah jurisprudence is perfunctory, and the empirical assertions are unverified. The structure recycles the same talking points across multiple sections. The references include items that are inconsistently formatted and, in some cases, appear non-verifiable. 

 The topic is timely and potentially significant, yet the manuscript adds little beyond what is already available in ministry summaries and practitioner alerts. The contribution is pitched as a doctrinal and comparative advance, but the paper does not deliver the close textual analysis or the analytical comparison that would differentiate it from a well-prepared practitioner memo. Assertions that the reforms will improve transparency, reduce litigation, and attract FDI are presented as conclusions rather than as findings grounded in evidence.

 The stated doctrinal method is not executed. A doctrinal analysis requires careful exegesis of the statutory text and implementing regulations, engagement with interpretive canons, and demonstration of how courts have read parallel provisions in analogous contexts. Here, article numbers are mentioned episodically without quotation, parsing, or systematic interpretation. The paper does not specify its primary corpus, the period of analysis, or the inclusion and exclusion criteria for judicial materials and ministerial circulars. There is no effort to map how the new provisions differ from the earlier regime beyond broad characterizations. The absence of case law analysis is conspicuous and fatally undermines the doctrinal claim.

 The comparison with the UAE and the UK is almost entirely descriptive. A serious comparative inquiry must explain why specific features travel, how they are adapted, and what institutional constraints shape selective borrowing. The manuscript does not address path dependence, enforcement capacity, or political-economy trade-offs. It asserts hybridization without explaining the mechanics of transplant and adaptation, and it overlooks granular issues such as the UK’s indemnity versus compensation framework under Regulation 17 and the UAE’s administrative approval practices for termination disputes. Without an explanatory framework, the comparative section remains a list of similarities and differences rather than analysis.

 There is no theoretical scaffolding. A paper of this kind must engage with legal transplant theory, institutional adaptation, or law-and-development critiques. It should articulate propositions about what tends to travel across systems, where friction occurs, and why. The text assumes that importing “best practices” will yield better outcomes and does not test this assumption against legal culture, adjudicative capacity, or administrative discretion. In the absence of a framework, the manuscript remains a narrative of reform rather than an academic study of reform.

 The integration of Shariah principles is cursory. Terms such as *gharar*, *fasakh*, and *akl al-māl bil-bāṭil* are invoked but not applied to concrete contractual clauses or litigated fact patterns. The paper does not present worked examples of Saudi judicial reasoning that reconcile statutory text with Shariah-based objections, particularly in areas such as agency termination, compensation, exclusivity, and arbitral award enforcement under public policy. Without case-based analysis and doctrinal reasoning, the claim to preserve an Islamic legal identity remains rhetorical.

 The manuscript makes multiple empirical assertions regarding FDI inflows, SCCA caseload growth, and digitisation outcomes but provides no reproducible data, tables, or sources. Vague phrases such as early indicators and data suggest appear repeatedly. The statement that no data are associated with the article is unacceptable given the extent of empirical claims. At minimum, the authors must furnish time-series data with sources, variable definitions, and time windows or retract the claims and recast them as hypotheses.

 The manuscript is bloated by repetition. The same points regarding arbitration, digital filing, and Vision 2030 recur across the introduction, the reform description, the FDI section, and the conclusion. Sentences are often long and imprecise, and paragraphs drift without analytical payoff. The paper could be shortened substantially without losing substance, and the saved space should be redeployed to rigorous doctrinal parsing and case analysis.

 Citation practice is inconsistent and, in places, questionable. Statutory references oscillate between different Hijri years and formats without official source details. Several references appear to be grey literature or are not readily verifiable, and Islamic legal terminology is not consistently italicised or transliterated. The manuscript needs a complete bibliographic audit, with official gazette citations for statutes, standard forms for case law, persistent identifiers for reports, and consistent treatment of Arabic terms.

 To become suitable for indexing, the manuscript must be rebuilt on a different foundation. The methods must define the primary sources, time frames, and analytic approach. The doctrinal analysis must quote and interpret the operative provisions and map them against prior law. The comparative section must be reoriented around an explanatory framework that accounts for selective borrowing and institutional constraints. The Shariah discussion must be anchored in concrete jurisprudence and doctrinal reasoning. Empirical statements must be supported by reproducible data or removed. The references must be verified and reformatted consistently. The structure must be tightened to remove repetition and sharpen the argument.

 I do not recommend indexing in its current form. The paper requires a fundamental redesign with substantial new analysis and verifiable evidence before it can be considered a credible scholarly contribution.

Is the work clearly and accurately presented and does it cite the current literature?

Partly

If applicable, is the statistical analysis and its interpretation appropriate?

Not applicable

Are all the source data underlying the results available to ensure full reproducibility?

Partly

Is the study design appropriate and is the work technically sound?

Partly

Are the conclusions drawn adequately supported by the results?

Partly

Are sufficient details of methods and analysis provided to allow replication by others?

No

Reviewer Expertise:

Political economy and Islamic Finance.

I confirm that I have read this submission and believe that I have an appropriate level of expertise to state that I do not consider it to be of an acceptable scientific standard, for reasons outlined above.

# Response

**Published:** 2025-11-10

**Authors:** Hajed A. Alotaibi

**Affiliations:** Majmaah University, Saudi Arabia

Dear Editorial Team,

 Many thanks for sharing the second peer-review report and for the clear guidance. I appreciate the reviewers’ thorough engagement with our paper. We have now prepared a revised version. In particular,

 1-we have: in the section of: Methodology and Sources, added: “To ensure methodological transparency, the corpus of legal texts analyzed covers the period 2018–2024, capturing both pre-reform and post-reform practice. Inclusion criteria encompassed all circulars and ministerial directives publicly issued under the Ministry of Commerce’s digital portal, as well as secondary literature providing interpretive context. Exclusion criteria omitted unpublished or inaccessible administrative guidance.

 The doctrinal analysis proceeds clause-by-clause, quoting operative statutory text where appropriate and interpreting it in light of Shariah maxims (qawāʿid fiqhiyyah) and comparative statutory equivalents. The analysis pays particular attention to how Saudi courts reconcile modern statutory provisions with Shariah principles such as gharar (uncertainty), fasakh (rescission), and akl al-māl bil-bāṭil (unjust enrichment). The comparative dimension employs a purposive sampling of the UAE Commercial Agency Law (Federal Law No. 3 of 2022) and the UK Commercial Agents (Council Directive) Regulations 1993. Comparative evaluation follows a three-step logic: (1) identify statutory parallels, (2) analyze their adaptation to local institutional constraints, and (3) evaluate cross-system transferability. The theoretical framing draws from legal transplant and institutional adaptation theories, explaining why particular reforms were selectively borrowed and how they interact with Saudi Arabia’s Shariah-based adjudicative culture. This allows the paper to move beyond description toward explaining how and why specific features travel and succeed”.

 2-Strengthen the doctrinal analysis by closely interpreting the operative provisions and mapping them systematically against the prior regime (with a concise side-by-side table to show what changed and why).

 3-Reframe and deepen the comparative component with a short “Comparative Framework” (legal transplants/institutional adaptation), and expand the analysis beyond description to explain how and why specific features travel or face friction, including enforcement capacity and institutional constraints.

**Topic**

**Pre-Reform Provision (1962 Law)**

**Reformed Provision (2022–2023 Law)**

**Doctrinal / Practical Effect**

 Registration

 Limited to Saudi nationals only; manual filing required

 Allows certain mixed-ownership entities; fully digital registration

 Broadens participation; reduces administrative delay

 Termination

 Vague criteria; heavy bias toward agent compensation

 Explicit grounds: expiry, breach, mutual consent; defined compensation

 Aligns with fasakh and gharar doctrines; enhances predictability

 Dispute Resolution

 Courts only; arbitration not recognized

 Arbitration expressly permitted (Art. 14); SCCA included

 Introduces enforceable ADR aligned with Vision 2030

 Transparency

 No public registry

 Online registry with periodic compliance reporting

 Promotes oversight and market trust

 4-Substantively integrate Sharia principles with worked examples that apply key doctrines to representative clauses (e.g., termination, compensation, exclusivity), and incorporate case-based reasoning where available.

 5-Handle empirical assertions responsibly by either (a) providing reproducible, sourced time-series data (with variable definitions and windows) in an appendix and a clear Data Availability statement, or (b) recasting such statements as hypotheses where data are insufficient.

 6-Tighten structure and prose to remove repetition, sharpen the argument, and keep “findings” and “discussion” either clearly distinguished or explicitly integrated with clear signposting.

 7-Conduct a full bibliographic audit to standardize statutory/case citations, provide official source details and persistent identifiers, and ensure consistent transliteration/italics for Arabic terms.

 Thank you again for the constructive feedback. We’re grateful for the reviewers’ time and these revisions.

 Best regards,

 Hajed

 (on behalf of the author)

# Reviewer report: Reviewer response for version 1

**DOI:** 10.5256/f1000research.186220.r414337

**Published:** 2025-09-23

**Authors:** Farhaan Wali

**Affiliations:** 1 Bangor University, Bangor, UK

**Overall Assessment**

 This is a strong, well-researched paper that makes a timely and significant contribution to the study of legal reform in Saudi Arabia. It situates the 2022–2023 reforms of the Commercial Agency Law within the wider context of *Vision 2030*, Shariah principles, and comparative international frameworks. The doctrinal and comparative approach is appropriate, and the paper succeeds in linking statutory analysis with economic and policy dimensions. The clarity of the abstract and the breadth of the literature used are commendable. However, there are areas where the paper could be improved before indexing. These include:

 Strengthening theoretical framing (beyond doctrinal analysis). Tightening comparative analysis for deeper insight (not just descriptive). Enhancing empirical grounding (current evidence is promising but thin). Refining structure to reduce repetition. Correcting style, consistency, and referencing.

**Major Corrections**

**Theoretical Framework**: The paper relies heavily on doctrinal and comparative legal analysis. While this is robust, it could benefit from engagement with **theoretical frameworks in legal reform studies** (e.g., legal transplant theory, institutional adaptation, or law-and-development scholarship). For example: What are the risks of “legal transplants” from UK or UAE law into a hybrid Shariah-based system? How do these reforms reflect broader debates on globalization vs. legal particularism?

**Comparative Analysis Depth: **The UAE and UK comparisons are insightful but remain largely **descriptive**. The authors should move beyond outlining similarities/differences to analysing *why* Saudi Arabia adopted certain features and not others Suggested expansion: discuss the **political economy of reform** (e.g., Vision 2030’s FDI goals) that shapes selective borrowing of foreign models.

**Empirical Support**: While references to FDI inflows, SCCA case statistics, and OECD/World Bank rankings are included, the evidence is **limited and anecdotal**. Recommendation: incorporate more **quantitative data** (e.g., comparative FDI inflows pre/post reform, number of agency registrations, World Bank “Ease of Doing Business” indicators). Even if full datasets are not available yet, clearer baseline data would strengthen the claims.

**Shariah Integration**: The paper acknowledges Shariah principles (e.g., gharar, fasakh) but treatment is somewhat cursory. Greater clarity is needed on: How Saudi judges are likely to reconcile statutory provisions with Shariah doctrines. Examples of jurisprudence where Shariah-based objections have shaped commercial law outcomes. Without this, the “Islamic legal identity” argument risks remaining underdeveloped.

**Repetition and Structure**: The paper repeats points (e.g., arbitration recognition, digitalisation, Vision 2030 objectives) across multiple sections (results, comparative analysis, FDI impact, and challenges). Recommendation: streamline to avoid redundancy and sharpen the argument in each section.

**Policy Recommendations**: The recommendations are comprehensive but read more like a **policy memo** than an academic conclusion. To align with scholarly standards, they should be more **analytically linked to the paper’s findings** rather than presented as a checklist. Suggest grouping into thematic clusters (e.g., judicial capacity, regulatory transparency, regional harmonisation).

**Minor Corrections**

**Style and Grammar**: Some sentences are overly long and complex. Shortening them would improve readability. Example: “Collectively, these statutory and practical deficiencies formed the impetus for reform, as policymakers recognized that the old regime was increasingly incompatible with the objectives of Vision 2030 and global trade norms” → could be split into two sentences.

**Consistency**: Inconsistent referencing of statutes (e.g., “Royal Decree No. M/11 of 1444H” vs. “Royal Decree No. M/11 of 1382H (1962)”). These need a consistent citation format throughout. Ensure uniformity in citing Islamic terms: e.g., *gharar* and *fasakh* should be italicised consistently.

**Referencing**: Some references appear **invented or unverifiable** (e.g., “Al-Zahrani, 2024”; “Alayed et al., 2025”). Authors should ensure accuracy and avoid future-dated citations unless these are genuine forthcoming works. A reference list was not provided with the manuscript — this must be included for peer review.

**Terminology**: Phrases like “hybridization” could be explained more clearly for an international audience unfamiliar with mixed legal systems. Clarify technical terms on first use (e.g., “fasakh” = rescission, “akl al-mal bil-batil” = unjust enrichment).

**Abstract**: The abstract is strong but slightly long. It could be condensed for sharper focus on methods, key findings, and implications.

**Formatting**: Subheadings are appropriate, but sometimes lengthy. Consider trimming: e.g., “Legal framework before the reforms” → “Pre-reform legal framework.”

**Recommendation**

**Revise and Resubmit (Major Revisions Needed)**

 The paper is promising and relevant to both legal scholarship and policy audiences. With tighter theoretical framing, stronger comparative insights, clearer Shariah integration, and removal of repetition, it will be well-positioned for indexing.

Is the work clearly and accurately presented and does it cite the current literature?

No

If applicable, is the statistical analysis and its interpretation appropriate?

Yes

Are all the source data underlying the results available to ensure full reproducibility?

Yes

Is the study design appropriate and is the work technically sound?

Yes

Are the conclusions drawn adequately supported by the results?

Yes

Are sufficient details of methods and analysis provided to allow replication by others?

Yes

Reviewer Expertise:

Islamic Studies, Islam in Britain, Islamism and radicalisation.

I confirm that I have read this submission and believe that I have an appropriate level of expertise to state that I do not consider it to be of an acceptable scientific standard, for reasons outlined above.

# Response

**Published:** 2025-09-24

**Authors:** Hajed A. Alotaibi

**Affiliations:** Majmaah University, Saudi Arabia

1) Response to Reviewer 1 

 We sincerely thank the Reviewer for the thoughtful and constructive report. We are encouraged by the assessment that the article is timely, well-researched, and policy-relevant. Below we respond point-by-point and indicate the precise revisions we have made (or will make) in the manuscript. Line/section references correspond to the revised text included in our resubmission. We have therefore updated our manuscript accordingly. Thank you.

 Major Corrections

 1) Theoretical framework

 Comment: Engage theories of legal reform (e.g., legal transplants, institutional adaptation, law-and-development).

 Response: Done. We added a new “Conceptual framing” segment at the end of the Introduction and cross-referenced it in Methodology. This section situates the reforms within legal transplant theory, institutional adaptation, and law-and-development debates, and explains tensions between globalization and legal particularism in a Shariah-based hybrid system. (See Introduction “Conceptual framing,” and Methodology last paragraph.)

 2) Comparative analysis depth

 Comment: Move beyond description; explain why KSA adopted some features (and not others), including political-economy (Vision 2030/FDI).

 Response: Done. The Comparative Analysis section now includes a new subpart “Why these features? A political-economy reading of selective borrowing” that links choices on arbitration, digitalization, and agent protections to Vision 2030 objectives and stakeholder incentives. (See Comparative Analysis, new subpart.)

 3) Empirical support

 Comment: Current FDI/SCCA data are promising but anecdotal; baseline and quantitative context are needed.

 Response: We will strengthen empirical grounding by including:

 Comparative FDI inflows (2018–2022, pre-reform vs. post-reform) from the General Authority for Statistics. Number of agency registrations digitized in 2023 (from Ministry of Commerce). World Bank Ease of Doing Business “Trading Across Borders” and “Starting a Business” indicators. Please see Impact on Foreign Direct Investment (FDI), Third section).

 4) Shariah integration

 Comment: Deepen analysis of how judges may reconcile statutory text with Shariah; provide jurisprudential examples.

 Response: Done. We added Section 6.1 “Judicial reconciliation with Shariah doctrines” with doctrinal anchors (gharar, fasakh, akl al-māl bil-bāṭil, maqāṣid al-sharīʿa) and illustrative jurisprudence (including the Board of Grievances example already referenced), and we explain interpretive techniques likely to be applied under the new law. Islamic terms are now italicized and defined on first use. (See 6.1.)

 5) Repetition and structure

 Comment: Streamline recurring mentions of arbitration, digitalization, Vision 2030 across sections.

 Response: Done. We consolidated: (i) all digitalization detail under The 2022–2023 Reforms; (ii) arbitration under Reforms + a concise link in Comparative Analysis; and (iii) Vision 2030 context centralized in Introduction with brief cross-references elsewhere.

 6) Policy recommendations

 Comment: Too checklist-like; link analytically and group thematically.

 Response: Done. We re-structured the Policy Recommendation Summary into three clusters—(1) judicial & institutional capacity, (2) regulatory transparency & compliance tooling, (3) regional harmonization & investor engagement—and tied each to specific findings in the paper.

 Minor Corrections

 Style: We split long sentences and reduced wordiness in the Introduction and Pre-reform sections.

 Consistency: Statutes now follow a uniform format (e.g., Royal Decree No. M/11 of 1382H (1962); Royal Decree No. M/11 of 1444H (2022–2023)).

 References: We verified entries and flagged obviously future-dated or non-verifiable items for replacement; Islamic legal terms are italicized consistently.

 Abstract: Reduced by ~60 words, sharpening methods/findings/implications.

 Formatting: Shortened subheadings (e.g., “Pre-reform legal framework”).

 We appreciate the Reviewer’s guidance; we believe these revisions materially strengthen the article’s analytical contribution and readability. 

 Corresponding author: Hajed A. Alotaibi (h.alotaibi@mu.edu.sa)
